# One-step estimation of networked population size: Respondent-driven capture-recapture with anonymity

**DOI:** 10.1371/journal.pone.0195959

**Published:** 2018-04-26

**Authors:** Bilal Khan, Hsuan-Wei Lee, Ian Fellows, Kirk Dombrowski

**Affiliations:** 1 Department of Sociology, University of Nebraska-Lincoln, Lincoln, Nebraska, United States of America; 2 Fellow Statistics, San Diego, California, United States of America; East China Normal University, CHINA

## Abstract

Size estimation is particularly important for populations whose members experience disproportionate health issues or pose elevated health risks to the ambient social structures in which they are embedded. Efforts to derive size estimates are often frustrated when the population is hidden or hard-to-reach in ways that preclude conventional survey strategies, as is the case when social stigma is associated with group membership or when group members are involved in illegal activities. This paper extends prior research on the problem of network population size estimation, building on established survey/sampling methodologies commonly used with hard-to-reach groups. Three novel one-step, network-based population size estimators are presented, for use in the context of uniform random sampling, respondent-driven sampling, and when networks exhibit significant clustering effects. We give provably sufficient conditions for the consistency of these estimators in large configuration networks. Simulation experiments across a wide range of synthetic network topologies validate the performance of the estimators, which also perform well on a real-world location-based social networking data set with significant clustering. Finally, the proposed schemes are extended to allow them to be used in settings where participant anonymity is required. Systematic experiments show favorable tradeoffs between anonymity guarantees and estimator performance. Taken together, we demonstrate that reasonable population size estimates are derived from anonymous respondent driven samples of 250-750 individuals, within ambient populations of 5,000-40,000. The method thus represents a novel and cost-effective means for health planners and those agencies concerned with health and disease surveillance to estimate the size of hidden populations. We discuss limitations and future work in the concluding section.

## 1 Introduction

Estimating the size of hidden and hard-to-reach populations is of critical importance to health officials seeking to mitigate the extent of health problems that may be concentrated within such populations [1], or when “reservoirs” of infection among a hidden population pose a health risk to the ambient population in which the hidden population is embedded [2, 3]. In the former, otherwise treatable maladies can remain unaddressed, multiplying eventual treatment costs when cases are discovered at more advanced stages. Such is the situation, for example, with mental illness among homeless and street dwelling populations [4–6]. An embedded “hidden” population can also frustrate intervention efforts that might otherwise be effective in the ambient population, preventing control of infection prevalence [7]. One example of this is the high prevalence of sexually transmitted disease among commercial sex workers [8–10]. In all such situations, health officials seek to estimate both the overall prevalence levels of maladies within a hidden population *and the size of the population itself*, in order to know the scope of treatment needs and overall social risk.

Efforts to ascertain prevalence and size estimates are frustrated by a range of factors that contribute to the “hiddenness” of the population. Such factors include heavy social stigma that inhibits the members of the hidden population from revealing their membership status. This is the case for people who inject drugs (PWID), who may be unwilling to self-identify as such under ordinary survey conditions [11, 12]. Hiddenness due to stigma can be further compounded when such activities are illegal, when they carry heavy personal costs (such as when self-identified heterosexual men also have sex with men), or when disease status is unknown (such as undiagnosed HIV infection rates among PWID). In these situations, conventional sampling is unreliable, and ordinary multiplier methods based on conventional sampling are rendered ineffective.

A number of techniques have been devised to address the problems of prevalence and population size estimation. These include capture-recapture [13, 14], chain referral [15, 16], venue-based sampling [17, 18], cluster sampling [19], and combinations thereof. Among the most popular is respondent-driven sampling (RDS) [20–22], which has been adapted for use in many situations, and which is employed widely in HIV surveillance efforts both within the United States and beyond [23]. RDS employs an incentivized chain referral process to recruit a sample of the hidden population. Under restricted but recognized conditions, RDS can be shown to result in a steady-state, “equilibrium” sample, and numerous methods have been derived for producing reasonable prevalence estimates from such a sample, while accounting for biases introduced in the referral process [24–29]. The ease of implementing RDS, the fact that it can operate under conditions of anonymity (via numbered coupons that track referrals), and its rigorous treatment under a range of statistical assumptions have made it a popular choice for researchers working with hidden populations [30]. While significant operational, design and analytical challenges frequently arise in deploying the RDS framework [31–33], the ability of the RDS-based methods to produce meaningful prevalence data remains, and presents considerable potential for use in population size estimation. Unfortunately, rigorous strategies for estimating the overall size of the hidden population from RDS data have been less successful, relying on simulation-based validation that fails to yield analytic insight, and generating widely varying estimates [34, 35]. While Berchenko and Frost have developed techniques that combine capture-recapture methods with RDS, their approach requires an initial degree-biased random sample and a second (independent) respondent driven sample [36]. Their hybrid schemes have been validated through simulations, and applied in the context of several field studies [37, 38]. In comparison, the approach we develop here requires only a single RDS sample, and is evaluated through both mathematical proofs and simulation experiments.

Other specialized methods have been developed to address size estimation for hidden populations, including capture-recapture procedures (sometimes called mark-recapture or multiplier procedures) [39, 40] and network scale-up methods (NSUM) [41]. Multiplier schemes typically use a sample of the hidden population and some external, often institutional knowledge-base (e.g. arrest records or hospital admissions) for estimation purposes [14, 42]. In these methods, two assumptions must generally be met: (i) the sample is representative of the hidden population at large, and (ii) everyone in the hidden population is equally likely to be “captured” in the official statistics being used [43]. While representativeness can sometimes be assumed (as in the case of RDS), it is often difficult to establish the uniformity of the capture statistics. Frankly stated, police arrests and hospital admissions can seldom be assumed to draw randomly from the hidden population. Further, capture-recapture/multiplier methods often require that the sample be identifiable in the institutional record, implying that expectations of anonymity on the part of sample respondents be abandoned. When working with hidden and highly stigmatized populations, such a sacrifice can be highly detrimental to both recruitment and informant reliability [44].

Network scale-up methods are also used to establish the size of hidden populations, though work in this area remains at an early stage. Here members of the entire population (ambient plus hidden) are asked to report on the number of known associates who fit the hidden population criteria [45, 46]. This approach has the advantage of being employable under ordinary random sampling conditions that can make use of known sampling frames (i.e. mail surveys and/or random digit dialing) [47]. However, the technique requires that ordinary people know whom among their associates fit the criteria for inclusion in the hidden category [48, 49]. Such an assumption faces objections in many of the situations in which we might wish to apply the technique, as when we seek to estimate the size of populations of PWID or sex workers. In these types of settings, individuals from the hidden population may go to great lengths to hide their membership status from friends and associates. Such effects inject “transmission error” into NSUM calculations, a quantity that is difficult to both detect and measure.

In previous work, we presented a novel capture-recapture methodology for estimating the size of a hidden population from an RDS sample [50], referred to there as the “telefunken” method. The method could be easily integrated into a conventional RDS framework, allowing researchers to continue to take advantage of the wide body of work on RDS and its ability to yield reliable prevalence estimates. The method was adopted experimentally in the context of efforts to collect data on commercially sexually exploited children [51] and, later, users of methamphetamine [52]. Both these studies made use of RDS and took place in New York City. Subsequent implementations of the technique provided further evidence of its effectiveness and ease of implementation [34]. The *telefunken* method was so named because its application entailed asking each RDS respondent to report on others in the population known to them by providing an encoding of their associates’ telephone number and demographic features (note that the technique is in no way related to the German apparatus company, Telefunken). In taking this approach, the method avoided reliance on official statistics (as needed in scale-up methods), and the requirement of drawing two independent samples (as needed by capture-recapture methods). Each individual’s code was created by considering a protocol-specified number of digits of their phone number, in order from last to first, and encoding each digit as 0/1 based on whether it was even or odd, and again 0/1 based on whether it was low (0-4) or high (5-9); in this manner, each subject and associate was “identified” by means of a multibit binary code. This many-to-one encoding allowed for ongoing anonymity for both respondents and their reported associates, while enabling the matching of contacts across numerous respondent interviews. In essence, the telefunken method represents a “one-step” approach which lifts many assumptions normally associated with other capture-recapture methods, and can be achieved using a single RDS sample from the hidden population. If shown to be effective, such an approach lends simplicity and greater cost-effectiveness to the size estimation procedure, potentially allowing for widespread application.

Concerning the issue of anonymity, independently and in roughly the same time period, Fellows put forward a general framework of Privatized Network Sampling (PNS) design [53]. PNS addresses two of the major concerns with regard to RDS data, namely the assumption that coupons are passed at random among alters, and that subjects can accurately report the number of alters that they have. As PNS is closely related to RDS, the standard RDS estimators may be used on data collected with a PNS design.

Given the growing interest in telefunken and PNS-like techniques [26, 34, 54], this paper aims to provide a systematic exposition of its strategy for one-step, anonymity preserving, network-based population size estimation. In what follows we formally describe the technique, analyze its mathematical properties, and validate its performance through simulations under a variety of implementation conditions. The simulations show considerable promise for the technique in scenarios normally associated with research among “hidden populations”. Limitations and next steps toward validation/extension are discussed at the end of the paper.

## 2 Background

Current network size estimation methods are based on quantifying the “repetition” or overlap observed across multiple samples [55]—where the category of objects sampled may be nodes, edges, distances, paths, motifs, or substructures [56, 57], depending on the specific approach in question.

- Node sampling methods often begin by taking independent uniform random samples of the population. In interpreting the overlap between samples [58, 59], these methods are based on the same principle as the well-studied “Coupon collector’s problem” from probability theory, for which maximum likelihood estimators and conservative confidence intervals are well known [60]. This classic method considers two uniform independent random samples [61]; in ecology, the method is often referred to as the “mark and recapture” protocol. Within a population *V*, the protocol first selects a uniform random “capture” sample *S* ⊆ *V*, and then a second (and independent) uniform random “recapture” sample *R* ⊆ *V*. From independence assumptions one infers that
  $$\frac{\left|V\right|}{\left|S\right|}\approx \frac{\left|R\right|}{\left|S\cap R\right|}$$ (1)
  and hence
  $$\left|V\right|\approx \frac{\left|S|\cdot |R\right|}{\left|S\cap R\right|}.$$ (2)
  The right-hand-side expression in (2) is known as the Lincoln-Peterson estimator [62, 63]. Many extensions and improvements to this classical technique have been developed, such as those making use of weighted sampling techniques [64], or sampling that is biased by the degree distribution of network nodes [65].
- Edge sampling approaches to population size estimation have also been developed [66–68]. These methods not only consider a sampled set of nodes, but also elicit a sample of their network neighbors. While edge sampling encounters problems associated with a bias toward high degree nodes, these methods offer potential gains in efficiency in dense graphs and where independent random sampling of nodes is restricted.
- Lastly, sampling via random walks represents a practical approach that is commonly used in estimating the size of social networks. Random walk methods start from an arbitrary node, then move to a neighboring node uniformly at random, and iterate. A typical random walk visits every node with a frequency proportional to its degree, but this bias can be quantified by Markov Chain analysis, and corrected to enable the derivation of an estimate of graph size from the frequency with which sampled nodes appear (and reappear) during the walk process. Random walk methods have largely used a sampling with replacement model, which may, in theory, introduce bias in estimates when the (fractional) size of the sample is large [24, 69]; however, there is some recent experimental evidence that such concerns may be overstated [70]. These methods are widely used to measure the size of online social networks, and are frequently employed in conjunction with a variety of web crawler data [71–75].

The approach developed here is inspired by and builds on several of the above strategies, including random walks and edge elicitation. An outline of this paper follows: In Section 3.1, we present a population estimator for uniform random samples. This estimator is extended for respondent-driven samples in Section 3.2. The two estimators are evaluated over a broad range of graph families (see Subsection 4.1) using a general experimental framework (see Subsection 4.2). The experimental results are presented in Sections 4.3 and 4.4. In Section 4.5, we adapt the estimators for use in networks with clustering, showing in Section 4.6 that the revised schemes continue to perform well on synthetic networks. In Section 5, we extend the network size estimation schemes to allow for protection of subject privacy. These anonymity-preserving extensions are evaluated through simulation experiments in Sections 5.2 and 5.3. The impact of non-uniformities is assessed in Section 6, with special consideration of degree bias in RDS seed selection, and bottlenecking due to community structure. The performance of the proposed estimators is evaluated on a real-world network in Section 7. Finally, discussion and limitations are presented in Section 8.

## 3 New population size estimators

We seek to generalize the Lincoln-Peterson framework of overlapping capture and recapture sets (2) to the context of networked populations, and describe it formally in the language of graphs. The following definition provides graph-theoretic notations which will be necessary in order to precisely define the proposed sampling and estimation processes.

**Definition 1**. *Let* *G* = (*V*, *E*) *be a graph*. *For each* *v* ∈ *V*, *denote the degree of v in G as* *d*(*v*). *Given* *A* ⊆ *V*, *denote the (arithmetic) mean degree of vertices in A as*:

$$\bar{d}(A)≔\frac{1}{\left|A\right|}\sum_{v\in A}d\left(v\right)$$ (3)

*and the (harmonic) mean degree of vertices in A as*

$$\tilde{d}(A)≔\frac{\left|A\right|}{\sum_{v\in A}\frac{1}{d(v)}}.$$ (4)

*noting that the latter is more robust against the presence of high-degree outliers*. *If* *H* = (*S*, *F*) *is a subgraph on* *S* ⊆ *V* *with edge set* *F* ⊆ *E* ∩ (*S* × *S*), *the “free neighborhood” of u (in G modulo H) is defined as*

N(u,F)≔{v|(u,v)∈E\F}⊆V. (5)

*Note that when G is allowed to have parallel edges (as is the case when it is obtained through configuration graph sampling), then* *N*(*u*, *F*) *may be a multiset. The “free ends” of S (in G modulo H) are taken to be the disjoint union (multiset)*

$$R(S,F)≔\underset{u\in S}{∐}N\left(u,F\right)\subseteq V$$ (6)

*and the “matches” of S (in G modulo H) are taken to be the disjoint union (multiset)*

$$M(S,F)≔\underset{u\in S}{∐}(N(u,F)\cap S)\subseteq V.$$ (7)

*We denote the respective cardinalities of these multisets as*

$$\begin{array}{cc} \langle R(S,F)\rangle ≔ & \sum_{u\in S}|N(u,F)|\\ \langle M(S,F)\rangle ≔ & \sum_{u\in S}|N(u,F)\cap S|. \end{array}$$

**Notation 1**. *In the arguments that follow, graph-theoretic quantities (such as those formalized in Definition 1) will sometimes be considered simultaneously in the context of more than one graph*—*e.g.* *G*_1_ = (*V*_1_, *E*_1_), *and* *G*_2_ = (*V*_2_, *E*_2_). *To avoid ambiguity in such settings, we will make the context clear by appending the graph as a parameter*—*e.g. the arithmetic mean degree of vertices in* *G*_1_ *is denoted* $\bar{d}\left(V_{1};G_{1}\right)$, *while the harmonic mean degree of vertices in* *G*_2_ *is expressed as* $\tilde{d}\left(V_{2};G_{2}\right)$.

**Notation 2**. *Whenever we are considering a multiset X, we will denote to its multiset cardinality as* 〈*X*〉, *while its set cardinality will be written as* |X*|. *For example, if* *X* = {1, 1, 2, 8, 8, 8} *then* 〈*X*〉 = 6, *while* |X*| = 3.

**Definition 2**. *Given multisets of vertices* *A*, *B* ⊆ *V* *we denote their characteristic functions as* $\chi_{A},\chi_{B}:V\to N$ *and define the multisets* *A*\*B*, *A* ∩ *B*, *A* ∪ *B* *by the respective characteristic functions*

$$\chi_{A\backslash B},\chi_{A\cap B},\chi_{A\cup B}:V\to N$$

*where for each* *v* ∈ *V*

$$\begin{array}{cc} \chi_{A\backslash B}(v)≔ & \text{max}\{0,\chi_{A}\left(v\right)-\chi_{B}\left(v\right)\}\\ \chi_{A\cap B}(v)≔ & \text{min}\{\chi_{A}\left(v\right),\chi_{B}\left(v\right)\}\\ \chi_{A\cup B}(v)≔ & \chi_{A}\left(v\right)+\chi_{B}\left(v\right). \end{array}$$

*We say that* *A* ⊆ *B* *are multisets*, *if* ∀*v* ∈ *V*, *we have* *χ*_*A*_(*v*) ≤ *χ*_*B*_(*v*).

### 3.1 Population size from a uniform random sample

With the formalisms of Definition 1 in place, we can define the estimator *n*_1_, which, given a uniform random subset of vertices *T* ⊆ *V*, yields an estimate of |*V*|.

**Definition 3**. *Given a graph* *G* = (*V*, *E*) *and T* ⊆ *V, define*

$$n_{1}(T)≔\frac{\left|T|\cdot \langle R(T,\emptyset )\right\rangle }{\left\langle M(T,\emptyset )\right\rangle }.$$ (8)

Lemma 1 shows that as the sample size grows, *n*_1_ converges to |*V*|.

**Lemma 1**. *Let* *G* = (*V*, *E*) *be a graph and let* *T*_1_ ⊆ *T*_2_ ⊆ *T*_3_ ⊆ … ⊆ *V* *be an ascending chain converging to* $\infty_{i=1}^{\infty }T_{i}=V$. *Then*

$$\underset{i\to \infty }{\text{lim}}\frac{n_{1}\left(T_{i}\right)}{\left|V\right|}=1.$$

*Proof*. Put *R*_*i*_ ≔ *R*(*T*_*i*_, ∅), *M*_*i*_ ≔ *M*(*T*_*i*_, ∅), and Δ_*i*_ ≔ *R*_*i*_\*M*_*i*_. Note that *R*_1_ ⊆ *R*_2_ ⊆ *R*_3_ ⊆ … and *M*_1_ ⊆ *M*_2_ ⊆ *M*_3_ ⊆ … are ascending chains of multisets, and *M*_*i*_ ⊆ *R*_*i*_ (*i* = 1, 2,…). Suppose *u* ∈ Δ_*i*_ and $\chi_{R_{i}}\left(u\right)=a$; clearly 0 < *a* ≤ *d*(*u*). Then since the ascending chain (*T*_*i*_)_*i* = 1, 2,…_ converges to *V*, there exists a least *j*_0_ > *i* for which $\chi_{M_{j}}\left(u\right)=d\left(u\right)$ and therefore $\chi_{\Delta_{j}}\left(u\right)=0$ for all *j* ≥ *j*_0_. It follows that

$$\overset{\infty }{\underset{i=1}{\cap }}R_{i}\backslash M_{i}=\emptyset$$

where multiset intersection and difference are as described in Definition 2, and thus

$$\underset{i\to \infty }{\text{lim}}\frac{\left\langle R_{i}\right\rangle }{\left\langle M_{i}\right\rangle }=1$$

which implies lim_*i*→∞_ *n*_1_(*T*_*i*_)/|*T*_*i*_| = 1, completing the proof.

The next proposition gives sufficient conditions under which uniform random samples *T* ⊆ *V* produce consistent estimates *n*_1_(*T*) ∼ |*V*| when |*V*| is large. Concrete realizations of these conditions are presented in Corollary 1.

**Proposition 1**. *For* *n* = 1, 2,… *let* *G*_*n*_ = (*V*_*n*_, *E*_*n*_) *be a graph on* |*V*_*n*_| = *f*(*n*) *vertices, where* *f*(*n*) *grows unboundedly*. *Let* *c*_*n*_ ∈ (0, 1] *and take T*_*n*_ ⊆ *V*_*n*_ *to be a subset of size* |*T*_*n*_| = ⌊*c*_*n*_ ⋅ *f*(*n*)⌋ *selected using uniform random sampling in* *V*_*n*_. *If* *c*_*n*_ ⋅ *f*(*n*) *diverges as n goes to infinity while*

$$c_{n}^{2}\cdot \bar{d}\left(V_{n}\right)⟶\Theta_{1}$$ (9)

*for some finite constant* Θ_1_ > 0, *then* $\frac{n_{1}\left(T_{n}\right)}{|V_{n}|}$ *necessarily converges to* 1.

*Proof*. Define random variables

$$\;\bar{\;R\;}{\;}_{n}≔\frac{1}{f(n)}\left\langle R\left(T_{n},\emptyset \right)\right\rangle =\frac{1}{f(n)}\sum_{u\in T_{n}}d\left(u\right)$$ (10)

$$\;\bar{\;M\;}{\;}_{n}≔\frac{1}{f(n)}\left\langle M\left(T_{n},\emptyset \right)\right\rangle .$$ (11)

For uniform random *u* ∈ *V*_*n*_, $E\left[d\left(u\right)\right]=\bar{d}\left(V_{n}\right)$. Since |*T*_*n*_| = ⌊*c*_*n*_ ⋅ *f*(*n*)⌋ diverges, the law of large numbers and linearity of expectation imply that as *n* tends to infinity

$$\left\langle R\left(T_{n},\emptyset \right)\right\rangle =\sum_{u\in T_{n}}d\left(u\right)\overset{p}{\to }\sum_{u\in T_{n}}\bar{d}\left(V_{n}\right)=\left|T_{n}\right|\cdot \bar{d}\left(V_{n}\right)$$ (12)

and thus

$$c_{n}\cdot \;\bar{\;R\;}{\;}_{n}=\frac{1}{f(n)}\left\langle R\left(T_{n},\emptyset \right)\right\rangle \overset{p}{\to }c_{n}\cdot \frac{1}{f(n)}\cdot \left|T_{n}\right|\cdot \bar{d}\left(V_{n}\right)=c_{n}^{2}\cdot \bar{d}\left(V_{n}\right)\overset{p}{\to }\Theta_{1}.$$ (13)

Now for each *u* ∈ *T*_*n*_ we have *E*[〈*N*(*u*, *F*_*n*_) ∩ *T*_*n*_〉] = *d*(*u*) ⋅ |*T*_*n*_|/*f*(*n*). Again, by the law of large numbers and linearity of expectation, as *n* tends to infinity

$$\;\bar{\;M\;}{\;}_{n}\overset{p}{\to }\;\bar{\;R\;}{\;}_{n}\cdot \frac{|T_{n}|}{f(n)}=\;\bar{\;R\;}{\;}_{n}\cdot c_{n}\overset{p}{\to }\Theta_{1}.$$ (14)

Considering (13) and (14) as preconditions of Slutsky’s theorem [76], we conclude:

$$\frac{n_{1}\left(T_{n}\right)}{f(n)}=\frac{1}{f(n)}\cdot \frac{c_{n}\cdot f\left(n\right)\cdot \;\bar{\;R\;}{\;}_{n}}{\;\bar{\;M\;}{\;}_{n}}\overset{d}{\to }\frac{{\text{plim}}_{n\to \infty }c_{n}\cdot \;\bar{\;R\;}{\;}_{n}}{{\text{plim}}_{n\to \infty }\;\bar{\;M\;}{\;}_{n}}=\frac{\Theta_{1}}{\Theta_{1}}=1.$$

The correspondence between Eq (8) in Definition 3 and our previous *telefunken* estimator is clear [77]. In addition, Eq (8) demonstrates a parallel structure with the Lincoln-Peterson estimator shown in expression (2): *T* represents the first assay (set); *R*(*T*, ∅) stands for the second assay (a multiset); the multiset *M*(*T*, ∅) is the subpopulation of the first assay that is recaptured by the second assay. Of course, in the present setting, the second assay *R*(*T*, ∅) is far from independent of the first assay *T*, since the two sets are intrinsically linked through the network geometry of *G*. Nevertheless, the fact that *T* is a random subset of *V* is enough to neutralize the impact of this non-independence and enable consistent estimation of population size.

**Corollary 1**. *Several special cases of Proposition 1 are of interest. In each of these cases, it is straightforward to verify that as n goes to infinity*, *c*_*n*_ ⋅ *f*(*n*) *diverges, while* $c_{n}^{2}\cdot \bar{d}\left(V_{n}\right)$ *tends to some finite strictly positive constant:*

- *When* *f*(*n*) = *O*(*n*), *c*_*n*_ = *O*(1) *is a constant, and* $\bar{d}\left(V_{n}\right)=O\left(1\right)$ *is a constant. In this case, we have a family of graphs of increasing size and constant average degree, in which we are taking uniform random samples whose size is a constant proportion of the entire population*.
- *When* *f*(*n*) = *O*(*n*), *c*_*n*_ = *O*(*g*(*n*)/*n*), *and* $\bar{d}\left(V_{n}\right)=O\left(n^{1-\epsilon }/g{\left(n\right)}^{2}\right)$, *where* *g*(*n*) *is a function which diverges, and* *ϵ* > 0 *is a constant. For example, if we take* *g*(*n*) = *n*^*ϵ*^, *then* *c*_*n*_ = *O*(1/*n*^1−*ϵ*^), *and* $\bar{d}\left(V_{n}\right)=O\left(n^{1-3\epsilon }\right)$. *As* *ϵ* *tends to 0, we approach a family of graphs of increasing size and linear average degree, in which we are taking uniform random samples of an absolute constant size. This special limit case is manifested by Erdős-Rényi graphs* [78].

### 3.2 Population size from a respondent-driven sample

Although the *n*_1_ estimator shows robust performance under uniform random sampling (see Section 4.3), random sampling is seldom a feasible strategy with hidden populations. As discussed above, sampling hard-to-reach populations presents considerable practical challenges [55], and many current surveys of hidden populations have come to depend on a tracked “peer referral” process known as respondent driven sampling [21].

For purposes of estimation, we consider a respondent-driven sample to be a random variable based on several parameters: an underlying networked population *G* = (*V*, *E*), a specified number of seeds |*D*|, the number of recruiting coupons *c* to be given to each subject, and the target sample size *r*. In our simulation experiments, the sampling procedure begins by randomly choosing |*D*| initial “seed” subjects in the network. For most of this paper, seeds are selected uniformly at random, though later, in Section 6, we will report on the differential impact of non-uniform RDS seed selection—specifically, seed selection that is biased by ego network size or restricted by the presence of community structures. Each seed subject is given *c* recruiting coupons and asked to participate in a “referral” process by distributing these among their study-eligible peers. Each subject *v* succeeds in recruiting between 0 and min{*c*, *d*(*v*)} individuals from their ego network, with the precise number being determined stochastically according to a specified distribution *δ*_*R*_ on {0, 1, …, *c*}. Each referred peer is assumed to come in for their interview at a time that is offset from their recruiter’s interview by an amount that random and exponentially distributed with rate λ_*W*_. When one or more of the recruited peers come in for interview with the coupon given to them by their recruiter, they too are given *c* coupons and asked to participate in the referral process. The scheme proceeds recursively in this manner using a finite number of 3*r* depletable coupons, until all *r* individuals have been recruited and interviewed. If (and whenever) the referral process stalls before *r* subjects have been interviewed, a new seed is recruited. Participation incentives are arranged to ensure that no subject will be the recipient of more than one coupon, and thus the process results in a collection of disjoint directed trees rooted at the seeds [79]. The precise values of the RDS parameters |*D*|, *c*, *r* and implementation parameters *δ*_*R*_, λ_*W*_ for our simulation experiments are detailed in Assumption 2; the stochastic process used to generate the underlying synthetic networks *G*(*V*, *E*) on which this RDS operates is described in Section 4.1.

Given the tendency of RDS to oversample high degree nodes, issues arise when estimation techniques attempt to make use of the degree statistics of a respondent driven sample. Special steps must be taken to account for differences between the average degree of an RDS sample and the average degree of the population from which the RDS sample is drawn. The simplifying assumption below is needed for our formal proofs of the proposed estimators’ performance. We emphasize that this assumption is not enforced (and is often violated) within the synthetic networks we used in our simulations, through which the proposed estimators’ performance was experimentally evaluated.

**Assumption 1**. *Whenever we are considering* *H* = (*S*, *F*) *to be a subgraph on* *S* ⊆ *V* *obtained through an RDS process inside graph* *G* = (*V*, *E*), *we will assume* $\tilde{d}\left(S\right)∼\bar{d}\left(V\right)$. *This assumption is justified in prior work* [20, 22], *is provably true for configuration graphs* [24], *and is reflective of the basic fact that the harmonic mean is robust against the presence of high-degree outliers, as we may expect to face when S is obtained via a non-uniform sampling process like RDS*.

The next estimator *n*_2_, provides an estimate |*V*| from a respondent driven sample *S* ⊆ *V*.

**Definition 4**. *Given a graph* *G* = (*V*, *E*), *a set* *S* ⊆ *V*, *and H* = (*S*, *F*) *a subgraph with edge set* *F* ⊆ *E* ∩ (*S* × *S*), *define*

$$n_{2}\left(S,F\right)≔\frac{\frac{\bar{d}\left(S\right)-1}{\tilde{d}\left(S\right)}\cdot \left|S\right|\cdot \left\langle R\left(S,F\right)\right\rangle }{\left\langle M(S,F)\right\rangle }$$ (15)

The next proposition gives sufficient conditions under which respondent-driven samples *S* ⊆ *V* produce consistent estimates *n*_2_(*T*) ∼ |*V*| when |*V*| is large.

**Proposition 2**. *For* *n* = 1, 2, … *let* *G*_*n*_ = (*V*_*n*_, *E*_*n*_) *be a graph obtained by configuration graph sampling via degree distribution* $D_{n}$, *where the vertex set size* |*V*_*n*_| = *f*(*n*) *grows unboundedly*. *Let* *c*_*n*_ ∈ (0, 1], *and take* *S*_*n*_ ⊆ *V*_*n*_ *to be a subset of size* |*S*_*n*_| = ⌊*c*_*n*_ ⋅ *f*(*n*)⌋ *selected using RDS sampling in* *G*_*n*_ *from* |*D*_*n*_| *seeds chosen uniformly at random. Define the random variable*

$$\Delta_{n}≔\frac{\bar{d}\left(S_{n}\right)-1}{\tilde{d}\left(S_{n}\right)}.$$

*Accepting Assumption 1*, *if* *c*_*n*_ ⋅ *f*(*n*)/*D*_*n*_ *diverges as n goes to infinity, while*

$$\Delta_{n}^{2}\cdot c_{n}^{2}\cdot \bar{d}\left(V_{n}\right)=\frac{{\left(\bar{d}\left(S_{n}\right)-1\right)}^{2}\cdot c_{n}^{2}}{\tilde{d}\left(S_{n}\right)}\overset{p}{\to }\Theta_{2}$$ (16)

*for some finite constant* Θ_2_ > 0, *then* $\frac{n_{2}\left(S_{n}\right)}{|V_{n}|}$ *necessarily converges to* 1.

*Proof*. Let (*S*_*n*_, *F*_*n*_) be a subgraph produced by an RDS sampling process in *G*_*n*_, and let *T*_*n*_ ⊆ *V*_*n*_ be an equal-sized set of vertices chosen by uniform random sampling, i.e. |*T*_*n*_| = |*S*_*n*_|. For random *u* ∈ *S*_*n*_ and *v* ∈ *T*_*n*_, as *n* tends to infinity

$$\frac{\left|N(u,\emptyset )\right|}{\bar{d}\left(S_{n}\right)}-\frac{\left|N(v,\emptyset )\right|}{\bar{d}\left(T_{n}\right)}=\frac{\left|N(u,\emptyset )\right|}{\bar{d}\left(S_{n}\right)}-\frac{\left|N(v,\emptyset )\right|}{\bar{d}\left(V_{n}\right)}=\frac{\left|N(u,\emptyset )\right|}{\bar{d}\left(S_{n}\right)}-\frac{\left|N(v,\emptyset )\right|}{\tilde{d}\left(S_{n}\right)}\overset{p}{\to }0.$$ (17)

where the first equality stems from the law of large numbers, and the second from Assumption 1. Now *S*_*n*_ is an RDS sample and hence is the disjoint union of *D*_*n*_ many trees. It follows that

$$\frac{|F_{n}|}{|S_{n}|}=1-\frac{|D_{n}|}{\left\lfloor c_{n}\cdot f\left(n\right)\right\rfloor }.$$

Since |*S*_*n*_| = ⌊*c*_*n*_ ⋅ *f*(*n*)⌋ diverges and *c*_*n*_ ⋅ *f*(*n*)/*D*_*n*_ diverges, we may conclude that

$$\underset{n\to \infty }{\text{lim}}\frac{|F_{n}|}{|S_{n}|}=1.$$ (18)

We note that |*N*(*u*, *F*_*n*_)| ≤ |*N*(*u*, ∅)|, and incorporating (18) back into the final expression in (17), we deduce

$$\frac{\left|N(u,F_{n})\right|}{\bar{d}\left(S_{n}\right)-1}-\frac{\left|N(v,\emptyset )\right|}{\tilde{d}\left(S_{n}\right)}\overset{p}{\to }0.$$ (19)

Definition 1’s Eq (6) and linearity of expectation then imply that as *n* tends to infinity

$$\left\langle R\left(S_{n},F_{n}\right)\right\rangle \overset{p}{\to }\frac{\bar{d}\left(S_{n}\right)-1}{\tilde{d}\left(S_{n}\right)}\cdot \left\langle R\left(T_{n},\emptyset \right)\right\rangle .$$ (20)

The configuration graph sampling process dictates that as *n* tends to infinity, for uniformly random *u* ∈ *S*_*n*_

$$E\left[\left\langle N\left(u,F_{n}\right)\cap S_{n}\right\rangle \right]=\left[\bar{d}\left(u\right)-1\right]\cdot \frac{\left\langle R\left(S_{n},F_{n}\right)\right\rangle }{2|E_{n}|}=\left[\bar{d}\left(u\right)-1\right]\cdot \frac{\left\langle R\left(S_{n},F_{n}\right)\right\rangle }{\bar{d}\left(V_{n}\right)\cdot f\left(n\right)}.$$

Definition 1’s Eq (7), expression (20), the law of large numbers, and linearity of expectation, together imply that as *n* tends to infinity

$$\left\langle M\left(S_{n},F_{n}\right)\right\rangle \overset{p}{\to }\frac{{\left\langle R\left(S_{n},F_{n}\right)\right\rangle }^{2}}{\bar{d}\left(V_{n}\right)\cdot f\left(n\right)}\overset{p}{\to }\frac{1}{\bar{d}\left(V_{n}\right)\cdot f\left(n\right)}\cdot [\frac{\bar{d}\left(S_{n}\right)-1}{\tilde{d}\left(S_{n}\right)}{]}^{2}\cdot {\left\langle R\left(T_{n},\emptyset \right)\right\rangle }^{2}.$$ (21)

Define the following random variables, closely related to (10) and (11) of Proposition 1:

$$R_{n}^{*}≔\left\langle R\left(S_{n},F_{n}\right)\right\rangle \;/\;f\left(n\right)=\Delta_{n}\cdot \;\bar{\;R\;}{\;}_{n}\overset{p}{\to }\Delta_{n}\cdot c_{n}\cdot \bar{d}\left(V_{n}\right)$$ (22)

$$M_{n}^{*}≔\left\langle M\left(S_{n},F_{n}\right)\right\rangle \;/\;f\left(n\right)=\Delta_{n}^{2}\cdot \;\bar{\;R\;}{\;}_{n}^{2}/\bar{d}\left(V_{n}\right)\overset{p}{\to }\Delta_{n}^{2}\cdot c_{n}^{2}\cdot \bar{d}\left(V_{n}\right)$$ (23)

From our assumptions on the convergence of $\Delta_{n}^{2}\cdot c_{n}^{2}\cdot \bar{d}\left(V_{n}\right)$, we see that as *n* tends to infinity

$$\Delta_{n}\cdot c_{n}\cdot R_{n}^{*}=\Delta_{n}^{2}\cdot c_{n}^{2}\cdot \bar{d}\left(V_{n}\right)\overset{p}{\to }\Theta_{2}$$ (24)

$$M_{n}^{*}\overset{p}{\to }\Theta_{2}$$ (25)

Considering (24) and (25) as preconditions of Slutsky’s theorem [76], we conclude:

$$\frac{n_{2}\left(S_{n}\right)}{f(n)}=\frac{1}{f(n)}\cdot \frac{\Delta_{n}\cdot c_{n}f\left(n\right)\cdot R_{n}^{*}}{M_{n}^{*}}\overset{d}{\to }\frac{{\text{plim}}_{n\to \infty }\Delta_{n}\cdot c_{n}\cdot R_{n}^{*}}{{\text{plim}}_{n\to \infty }M_{n}^{*}}=\frac{\Theta_{2}}{\Theta_{2}}=1.$$

## 4 Evaluating the *n*_1_ and *n*_2_ estimators

To evaluate the proposed estimators *n*_1_ (8) and *n*_2_ (15), we conducted simulation experiments on samples drawn from synthetic networks using uniform and respondent-driven sampling, respectively. Underlying networks were selected by configuration sampling techniques [80–82] relative to Lognormal, Poisson, and Exponential distributions. We also considered Barabási-Albert graphs [83], and Erdős-Rényi graphs [78].

### 4.1 Synthetic networks

The tendency of RDS to over-recruit high degree nodes is well known, and readily evidenced in experiments on idealized topologies. Attempts to model peer-referral or “snowball” recruitment processes point to the fact that the degree distribution of nodes can influence the performance of mean estimators [84], suggesting Bayesian approaches which make use of degree distribution data in the derivation of population size estimates [35, 85]. To validate the *n*_1_ and *n*_2_ estimators against a wide range of possible topologies, five idealized families of random graphs were used to perform initial experiments. In later sections, we take up the issue of clustering (Section 4.5), anonymity (Section 5), non-uniformity in the seed selection (Section 6), and performance on a real-world network (Section 7).

In what follows, configuration graphs were sampled (relative to a specified degree distribution) by first attaching the prescribed number of free half-edges to each node. Pairs of free half-edges were then chosen uniformly at random and bound together to form an edge, repeatedly, until no free half-edges remain. Note that this sampling process may yield graphs that have multiple parallel edges and self loops.

**Definition 5**. *Given a set* *V* *with* |*V*| = *n*, *for each* λ∈R, λ > 1, *let distributions* $D_{L(\lambda )}$, $D_{P(\lambda )}$, $D_{X(\lambda )}$, *and* $D_{R(\lambda )}:V\to N$ *be defined such that for each* *v* ∈ *V:*

- $D_{L(\lambda ,n)}\left(v\right)=1+X$ *where* *X* *is a Lognormal random variable with mean* λ − 1 *and standard deviation 1*.
- $D_{P(\lambda ,n)}\left(v\right)=1+X$ *where* *X* *is a Poisson random variable with rate parameter* λ − 1.
- $D_{X(\lambda ,n)}\left(v\right)=1+X$ *where* *X* *is an Exponential random variable with mean* λ − 1.

*Corresponding to each of the three distributions above, let* L(λ,n), P(λ,n), X(λ,n), R(λ,n) *be the sample spaces of configuration graphs* *G* = (*V*, *E*) *where* |*V*| = *n*. *Note that a random graph drawn from these sample spaces will have expected mean vertex degree* $E[\bar{d}\left(V\right)]=\lambda$.

**Definition 6**. *For each* λ∈R, λ > 1, *let* B(λ,n) *be the sample space of* *n*-*vertex Barabási-Albert graphs* *G* = (*V*, *E*). *Each such graph is the final output of a process which produces a sequence of graphs* *G*^*i*^ = (*V*^*i*^, *E*^*i*^) *on* *V*^*i*^ ≔ {*v*_1_, … *v*_*i*_} *with* λ ≤ *i* ≤ *n*. *The initial graph* *G*^λ^ = (*V*^λ^, *E*^λ^) *is taken to be the complete graph on* λ *vertices, i.e.* *E* = *V*^λ^ × *V*^λ^. *At each stage* *i* > λ *of the process, node* *v*_*i*_ (λ < *i* ≤ *n*) *connects to a random number*

$$\Delta_{i}≔|E_{i}\backslash E_{i-1}|=\{\begin{array}{cc} \left\lfloor \lambda /2\right\rfloor & \;with\;probability\;1+\lfloor \lambda \rfloor -\lambda \\ 1+\lfloor \lambda /2\rfloor & \;otherwise. \end{array}$$

*of pre-existing nodes* $\{p_{i,1},\ldots p_{i,\Delta_{i}}\}\subseteq V^{i-1}$. *This set is constructed by sequential sampling without replacement, i.e. as* *l* = 1, …, Δ_*i*_, *each of the candidates* *w* ∈ *C*_*i*, *l*_ ≔ *V*^*i*−1^\{*v*_*i*,1_, … *v*_*i*,*l*−1_} *is chosen with a probability that reflects degree-biased preferential attachment*

$$Prob\left(p_{i,l}=w\right)=\frac{1+d(w;G^{i-1})}{\sum_{w^{\prime }\in C_{i,l}}1+d\left(w^{\prime };G^{i-1}\right)}.$$

*Here* *d*(*w*; *G*^*i*−1^) *denotes the degree of vertex w in graph* *G*^*i*−1^ = (*V*^*i*−1^, *E*^*i*−1^). *The final member of the resulting sequence* *G*^*n*^ = (*V*^*n*^, *E*^*n*^) *is output as the sampled graph. Note that if* *n* ≫ λ, *the process above results in a graph* *G* = (*V*, *E*), *sampled from* B(λ,n), *and having expected mean vertex degree* $E[\bar{d}\left(V\right)]∼\lambda$.

**Definition 7**. *For each* λ∈R, λ > 0, *let* E(λ,n) *be the sample space of* *n*-*vertex Erdős-Rényi graphs* *G* = (*V*, *E*), *where* *E* ⊆ *V* × *V* *is a random subset constructed uniformly at random by taking:*

$$Prob\left(\left(u,v\right)\in E\right)=\{\begin{array}{cc} \lambda /(n-1) & u\neq v\\ 0 & u=v \end{array}$$

*for each* (*u*, *v*) ∈ *V* × *V*. *Note that a random graph* *G* = (*V*, *E*) *drawn from* E(λ,n) *will have expected mean vertex degree* $E[\bar{d}\left(V\right)]∼\lambda$.

### 4.2 Experimental framework

For each of the 5 families L(λ,n),P(λ,n),X(λ,n),B(λ,n), and E(λ,n) defined in Section 4.1, we varied λ = 3, 5, 10; from each of these 15 concrete sample spaces, we used configuration graph sampling to select 30 random graphs of sizes *n* = 5000, 10*K*, 20*K* and 40*K*. In each of these 5 × 3 × 4 × 30 = 1,800 graphs, we generated 30 uniform and 30 RDS samples of size *r* = 250, 500 and 750. In this manner, a total of 1, 800 × 30 × 3 × 2 = 324, 000 simulations were conducted. Section 4.3 reports on simulation experiments in which *n*_1_ was applied to uniform random samples; experiments in which *n*_2_ was applied to respondent driven samples are presented in Section 4.4.

### 4.3 Evaluating *n*_1_ on synthetic networks

**Fig 1:**
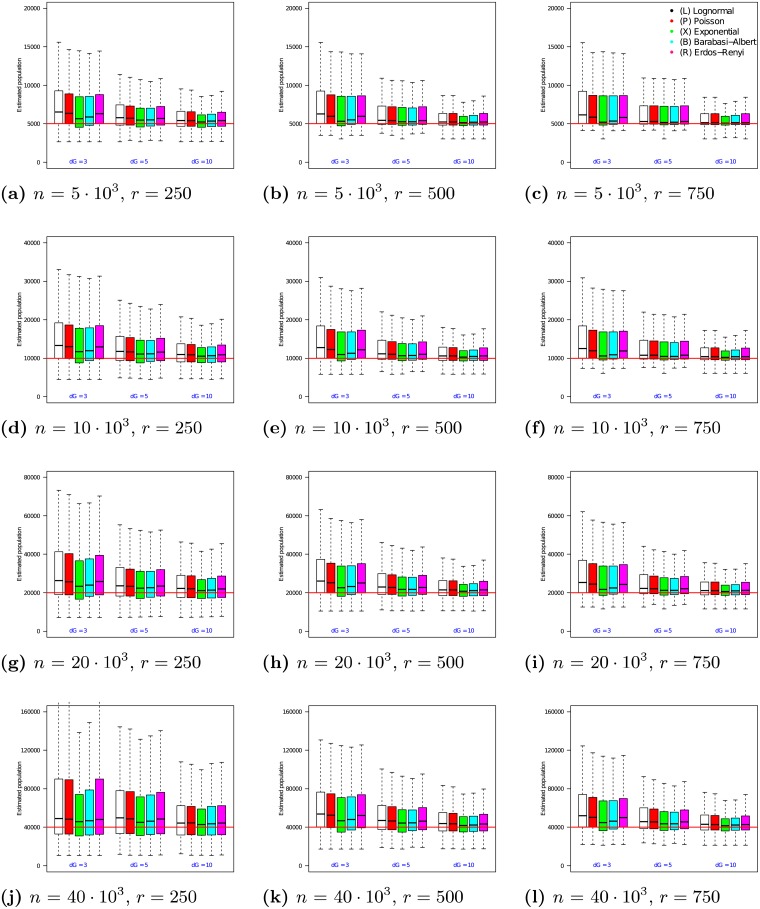
Estimator *n*_1_ on uniform samples in populations of size *n* = 5 ⋅ 10^3^ to 40 ⋅ 10^3^. In each box, the thick line indicates the sample median; the top of the box is the median of the upper half of the estimated values (75% quartile); the bottom of the box indicates the median of the lower half of the estimated values (25% quartile); and the whiskers indicate the full range of estimated values. No (finite) outliers were removed.

The experiments here follow the framework described in Section 4.2 and use uniform random samples. The 12 graphs in Fig 1 present the performance of the *n*_1_ estimator as the true population size *n* is varied from 5 ⋅ 10^3^ to 40 ⋅ 10^3^ (vertical axis of the grid) and the size of the uniform sample is varied from 250 to 750 (horizontal axis of the grid). In each of the 12 graphs, the x-axis varies the average degree λ from 3 to 10. For each choice of λ, the medians and quartile ranges of *n*_1_ are given for each of the 5 graph families. Each of these is determined by 900 simulations (30 graphs times 30 uniformly drawn samples in each graph).

Fig 1 shows that as sample size increases, the medians of *n*_1_ converge to the true population size. For example, when *n* = 5 ⋅ 10^3^ and *r* = 250, Exponential degree distribution graphs with λ = 3 have a median *n*_1_ value of 5663 (a 13.3% offset from the true value of *n* = 5 ⋅ 10^3^). In comparison, when *r* = 750, the median for this family of graphs is 5204 (just 4.1% offset from the true value). As the sample size increases from *r* = 250 to *r* = 750, the error in the median estimate decreases by 9.2%. The benefit of increasing sample size diminishes as networks grow larger, however. For example, for a network of size *n* = 40 ⋅ 10^3^, increasing the sample size from *r* = 250 to *r* = 750 causes the error in the median *n*_1_ estimate to undergo only a 2% change.

In addition, Fig 1 shows that as sample size increases, the interquartile range (IQR) of the estimates decreases. For example, when *n* = 5 ⋅ 10^3^ and *r* = 250, Lognormal degree distribution graphs with λ = 10 experience an interquartile range of 1950 in their *n*_1_ estimates (35.9% of the median). In comparison, when *r* = 750, the interquartile range for this family of graphs decreases to 1425 (a 26.9% reduction). The magnitude of this effect increases as networks grow larger. For example, for a network of size *n* = 40 ⋅ 10^3^, increasing the sample size from *r* = 250 to *r* = 750 causes the interquartile range of the *n*_1_ estimate to undergo a 48.6% decrease.

### 4.4 Evaluating *n*_2_ on synthetic networks

The experiments in this and all subsequent sections use respondent-driven samples. The precise values of the RDS parameters |*D*|, *c*, *r* and implementation parameters *δ*_*R*_, λ_*W*_ are given below.

**Assumption 2**. *In all our experiments where RDS is used to generate samples, we take* |*D*| = 7 *random seeds drawn uniformly at random from V. Each subject was given* *c* = 3 *coupons. Depending on the experiment, the sample size r was either 250, 500, or 750. Reflecting our experiences in the field* [86], *we took the recruiting success distribution* *δ*_*R*_ *such that each subject had a 90% chance of recruiting 2 subjects randomly from their ego network, and a 10% chance of recruiting just 1. [Individuals with an ego network of size 1 were assumed to recruit that one individual with 100% probability, while individuals with an ego network of size 0 recruited no one]. The delay between recruiter and recruited subjects’ interview times were assumed to be exponentially distributed with rate* λ_*W*_ = 1.

**Fig 2:**
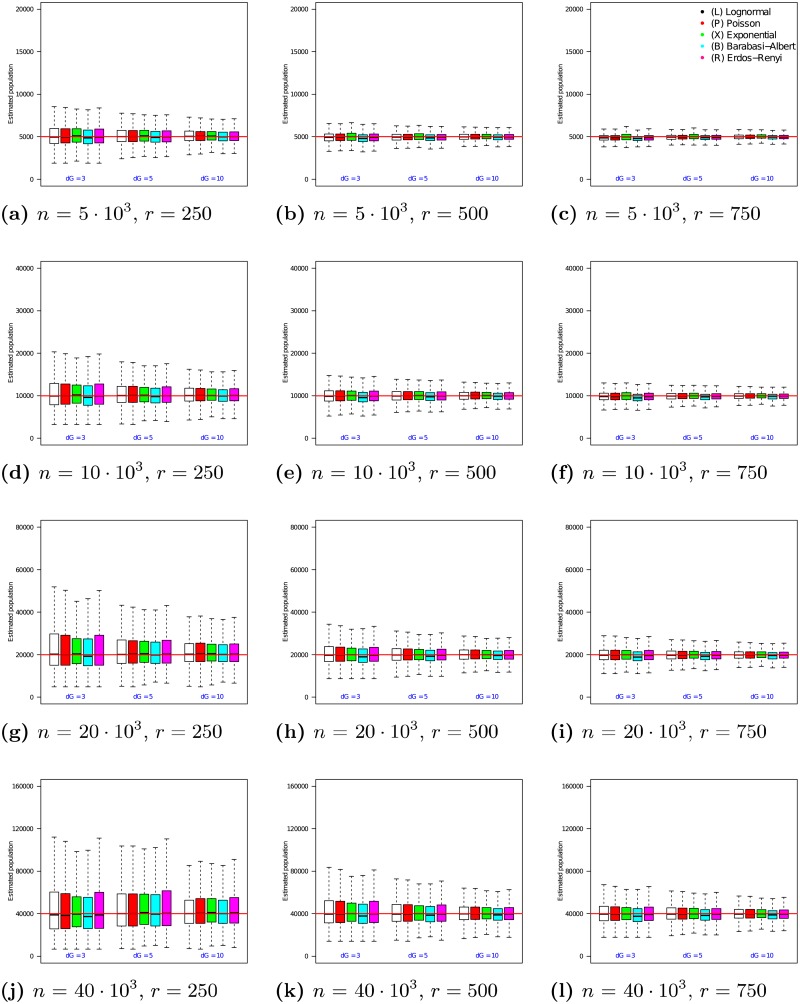
Estimator *n*_2_ on RDS samples in populations of size *n* = 5 ⋅ 10^3^ to 40 ⋅ 10^3^. In each box, the thick line indicates the sample median; the top of the box is the median of the upper half of the estimated values (75% quartile); the bottom of the box indicates the median of the lower half of the estimated values (25% quartile); and the whiskers indicate the full range of estimated values. No (finite) outliers were removed.

The 12 graphs in Fig 2 present the performance of the *n*_2_ estimator as the true population size *n* is varied from 5 ⋅ 10^3^ to 40 ⋅ 10^3^ (vertical axis of the grid) and the size of the RDS sample is varied from 250 to 750 (horizontal axis of the grid). In each of the 12 graphs, the x-axis varies the average degree λ from 3 to 10. For each choice of λ, the medians and quartile ranges of *n*_2_ are given for each of the 5 graph families. Each of these is determined by 900 simulations (30 graphs times 30 uniformly drawn samples in each graph).

Fig 2 shows that the median of *n*_2_ converges to the true population size across a range of topologies, RDS sample sizes, and overall populations. In addition, Fig 2 shows that as sample size increases, the interquartile difference decreases. For example, when *n* = 5 ⋅ 10^3^ and *r* = 250, Poisson degree distribution graphs with λ = 3 experience an interquartile range of 1676 in their *n*_2_ estimates (33.8% of the median). In comparison, when *r* = 750, the interquartile range for this family of graphs decreases to 524 (a 68.7% reduction). The magnitude of this effect decreases as networks grow larger, such that, for a network of size *n* = 40 ⋅ 10^3^, increasing the sample size from *r* = 250 to *r* = 750 causes the interquartile range of the *n*_2_ estimate to undergo a 60.8% decrease. However, the total range of estimates as a proportion of the median decreases as sample size increases, indicating decreasing sample-based variance (a key concern in RDS sampling [28]).

### 4.5 Population size estimation in the presence of clustering

Beyond the oversampling of high degree nodes, RDS faces challenges when used in networks where network clustering is pronounced [49, 87]. While methods are available to assess the presence of clustering [25], and recent work has proposed new techniques to estimate and account for clustering from a single RDS sample [88], the effects of this phenomenon on population size estimation from RDS samples is seldom discussed. The root of the problem lies in the fact that RDS walks necessarily sample network neighborhoods. Where neighbors show high levels of network transitivity, counts of common edges will produce high numbers of “matches” that appear in the denominator of both *n*_1_ and *n*_2_. This will bias the estimates of overall population size derived from these estimators toward underestimation of the total network size.

In the context of random walk techniques, one approach to this problem is to *only* consider collisions among nodes that are *far away* from each other in the sampling chain when inferring a population size estimate [75]. A similar approach is taken here by considering neighbor overlap among respondents whose path distances in the RDS chains are above a specific threshold. For simplicity, here we take this threshold to be infinity, leaving the consideration of finite thresholds for consideration in future research. In short, we consider a modification of *n*_2_ that discounts matched free ends within a single RDS sampling tree and, for purposes of estimation, only counts those matches that occur across distinct RDS trees. The next Definition introduces formalisms necessary to make this precise.

**Definition 8**. *Let* *G* = (*V*, *E*), *take* *S* ⊆ *V*, *and let* *H* = (*S*, *F*) *be a subgraph on* *S* ⊆ *V* *with edge set* *F* ⊆ *E* ∩ (*S* × *S*) *obtained by respondent driven sampling from a set of seeds* *D* ⊆ *S* *where* |*D*| > 1. *Define the function* *γ*: *S* → *D* *associating each* *u* ∈ *S* *with the unique seed* *γ*(*u*) ∈ *D from which u was discovered through a sequence of referrals. For each* *u* ∈ *S*, *the component of u is denoted*

$$C_{\gamma }(u)≔\{v\;|\;\gamma (v)=\gamma (u)\}\subseteq S$$ (26)

*while its complement is written* ${\tilde{C}}_{\gamma }\left(u\right)≔S\backslash C_{\gamma }\left(u\right)$. *Note that* $C_{\gamma }\left(u\right)\cap {\tilde{C}}_{\gamma }\left(u\right)=\emptyset$. *For each seed* *s* ∈ *D*, *we define the cross-seed matches from the* *C*_*γ*_(*u*) *component (in* *G* *modulo* *H*) *as the disjoint union (multiset)*

$$X(s,F,\gamma )≔\underset{u\in C_{\gamma }\left(s\right)}{∐}(N\left(u,F\right)\cap {\tilde{C}}_{\gamma }\left(s\right))\subseteq V$$ (27)

*whose cardinality is denoted*

$$\langle X(s,F,\gamma )\rangle ≔\sum_{u\in C_{\gamma }\left(s\right)}|N\left(u,F\right)\cap {\tilde{C}}_{\gamma }\left(s\right)|.$$

The next estimator *n*_3_, provides a revised estimate |*V*| from a respondent driven sample *S* ⊆ *V*, discounting matches that occur within the same RDS component.

**Definition 9**. *Given a graph* *G* = (*V*, *E*), *a set* *S* ⊆ *V*, *and H* = (*S*, *F*) *a subgraph on* *S* ⊆ *V* *with edge set* *F* ⊆ *E* ∩ (*S* × *S*). *Take* *D* ⊆ *S* *satisfying* |*D*| > 1 *and*

$$s_{1}\neq s_{2}\Rightarrow C_{\gamma }\left(s_{1}\right)\cap C_{\gamma }\left(s_{2}\right)=\emptyset .$$

*Define*

$$n_{3}(S,F,D,\gamma )≔\frac{\sum_{s\in D}\frac{\bar{d}({\tilde{C}}_{\gamma }(s))-1}{\tilde{d}(S)}\cdot |{\tilde{C}}_{\gamma }(s)|\cdot \langle R(C_{\gamma }(s),F)\rangle }{\sum_{s\in D}\langle X(s,F,\gamma )\rangle }.$$ (28)

The next proposition gives sufficient conditions under which respondent-driven samples *S* ⊆ *V* produce consistent estimates *n*_3_(*T*) ∼ |*V*| when |*V*| is large.

**Proposition 3**. *For* *n* = 1, 2, …, *let* *G*_*n*_ = (*V*_*n*_, *E*_*n*_) *be a graph on* |*V*_*n*_| = *f*(*n*) *vertices obtained by configuration graph sampling via degree distribution* $D_{n}$, *where* *f*(*n*) *grows unboundedly*. *Let* *c*_*n*_ ∈ (0, 1], *and take* *S*_*n*_ ⊆ *V*_*n*_ *to be a subset of size* |*S*_*n*_| = ⌊*c*_*n*_ ⋅ *f*(*n*)⌋ *selected using RDS sampling in* *G*_*n*_ *from* |*D*_*n*_| > 1 *seeds*. *Define the random variable*

$$\Delta_{n}≔\frac{\bar{d}\left(S_{n}\right)-1}{\tilde{d}\left(S_{n}\right)}.$$

*Accepting Assumption 1*, *if* *c*_*n*_ ⋅ *f*(*n*)/*D*_*n*_ *diverges as n goes to infinity, while*

$$\Delta_{n}^{2}\cdot c_{n}^{2}\cdot \bar{d}\left(V_{n}\right)\cdot \frac{|D_{n}|-1}{|D_{n}|}=\frac{{\left(\bar{d}\left(S_{n}\right)-1\right)}^{2}\cdot c_{n}^{2}}{\tilde{d}\left(S_{n}\right)}\cdot \frac{|D_{n}|-1}{|D_{n}|}\overset{p}{\to }\Theta_{3}$$ (29)

*for some finite constant* Θ_3_ > 0, *then* $\frac{n_{3}\left(S_{n},F_{n},D_{n},\gamma \right)}{f(n)}$ *necessarily converges to* 1.

*Proof*. Since each seed *s* ∈ *D*_*n*_ is chosen uniformly at random, and RDS recruits from all seeds concurrently, and |*S*_*n*_| = ⌊*c*_*n*_ ⋅ *f*(*n*)⌋ diverges, for random *s* ∈ *D*_*n*_, we know that

$$|C_{\gamma }\left(s\right)|\overset{p}{\to }\frac{1}{|D_{n}|}\cdot \left|S_{n}\right|=\frac{c_{n}\cdot f\left(n\right)}{|D_{n}|}$$ (30)

$$|{\tilde{C}}_{\gamma }\left(s\right)|\overset{p}{\to }\frac{|D_{n}|-1}{|D_{n}|}\cdot \left|S_{n}\right|=\frac{|D_{n}|-1}{|D_{n}|}\cdot c_{n}\cdot f\left(n\right)$$ (31)

$$\bar{d}\left(C_{\gamma }\left(s\right)\right),\;\;\bar{d}\left({\tilde{C}}_{\gamma }\left(s\right)\right)\overset{p}{\to }\bar{d}\left(S_{n}\right).$$ (32)

Combining (30) and (32), we conclude

$$\left\langle R\left(C_{\gamma }\left(s\right),F_{n}\right)\right\rangle \overset{p}{\to }\frac{\left\langle R\left(S_{n},F_{n}\right)\right\rangle }{|D_{n}|}.$$ (33)

Sufficient reasoning about the configuration graph construction process tells us

$$\left\langle X\left(s,F_{n},\gamma \right)\right\rangle \overset{p}{\to }\frac{1}{|D_{n}|}\cdot \left\langle M\left(S_{n},F_{n}\right)\right\rangle \cdot \frac{|D_{n}|-1}{|D_{n}|}.$$ (34)

Define the following random variables, closely related to (22) and (23) of Proposition 2:

$$\begin{array}{ll} R_{n}^{\circ }≔ & \sum_{s\in D_{n}}\frac{\bar{d}\left({\tilde{C}}_{\gamma }\left(s\right)\right)-1}{\tilde{d}\left(S\right)}\cdot \left|{\tilde{C}}_{\gamma }\left(s\right)\right|\cdot \langle R\left(C_{\gamma }\left(s\right),F_{n}\right)\rangle /f\left(n\right)\\ M_{n}^{\circ }≔ & \sum_{s\in D_{n}}\langle X\left(s,F_{n},\gamma \right)\rangle /f\left(n\right). \end{array}$$

As *n* tends to infinity

$$\begin{array}{l} R_{n}^{\circ }\overset{p}{\to }\frac{\bar{d}\left(S_{n}\right)-1}{\tilde{d}\left(S\right)}\left(\frac{|D_{n}|-1}{\left|D_{n}\right|}\cdot c_{n}\cdot f\left(n\right)\right)\cdot R_{n}^{*}\left(S_{n},F_{n}\right)\\ M_{n}^{\circ }\overset{p}{\to }\frac{|D_{n}|-1}{\left|D_{n}\right|}\cdot M_{n}^{*}\left(S_{n},F_{n}\right). \end{array}$$

where

$$R_{n}^{*}\left(S_{n},F_{n}\right)\overset{p}{\to }\Delta_{n}\cdot c_{n}\cdot \bar{d}\left(V_{n}\right)$$

as noted in (22), while

$$M_{n}^{*}\left(S_{n},F_{n}\right)\overset{p}{\to }\Delta_{n}^{2}\cdot c_{n}^{2}\cdot \bar{d}\left(V_{n}\right)$$

as noted in (23). Thus

$$\begin{array}{l} R_{n}^{\circ }\overset{p}{\to }\Delta_{n}^{2}\cdot c_{n}^{2}\cdot \bar{d}\left(V_{n}\right)\cdot \frac{|D_{n}|-1}{\left|D_{n}\right|}\cdot f\left(n\right)=\Theta_{3}\cdot f\left(n\right)\\ M_{n}^{\circ }\overset{p}{\to }\Delta_{n}^{2}\cdot c_{n}^{2}\cdot \bar{d}\left(V_{n}\right)\cdot \frac{|D_{n}|-1}{\left|D_{n}\right|}=\Theta_{3}. \end{array}$$

By Slutsky’s theorem [76], it follows that

$$\frac{n_{3}\left(S_{n},F_{n},D_{n},\gamma \right)}{f(n)}=\frac{\frac{1}{f(n)}\cdot R_{n}^{\circ }}{M_{n}^{\circ }}\overset{d}{\to }\frac{{\text{plim}}_{n\to \infty }\frac{1}{f(n)}\cdot R_{n}^{\circ }}{{\text{plim}}_{n\to \infty }M_{n}^{\circ }}=\frac{\Theta_{3}}{\Theta_{3}}=1.$$ (35)

### 4.6 Evaluating *n*_3_ on synthetic networks

**Fig 3:**
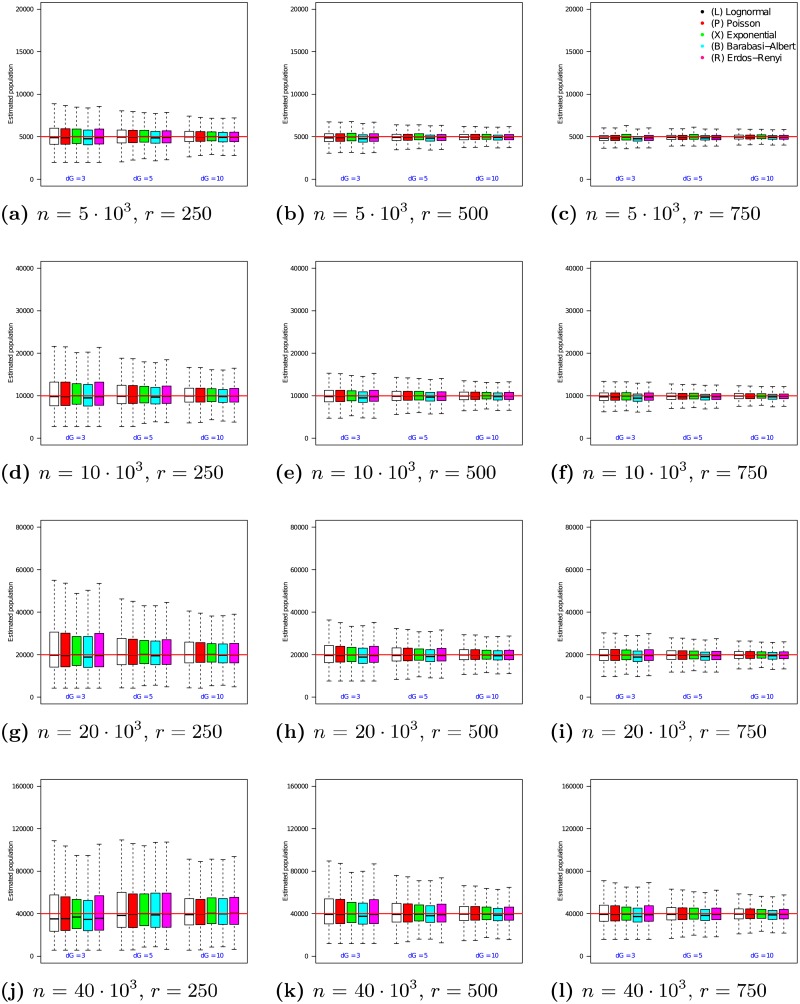
Estimator *n*_3_ on RDS samples in populations of size *n* = 5 ⋅ 10^3^ to 40 ⋅ 10^3^. In each box, the thick line indicates the sample median; the top of the box is the median of the upper half of the estimated values (75% quartile); the bottom of the box indicates the median of the lower half of the estimated values (25% quartile); and the whiskers indicate the full range of estimated values. No (finite) outliers were removed.

Prior to examining the performance of *n*_3_ on empirical networks, we first look at its performance on the synthetic networks used to evaluate *n*_1_ and *n*_2_. The experiments shown in Fig 3 follow the framework described in Section 4.2 and use respondent driven samples, each obtained via an RDS process operating as specified in Assumption 2.

The 12 graphs in Fig 3 present the performance of the *n*_3_ estimator as the true population size *n* is varied from 5 ⋅ 10^3^ to 40 ⋅ 10^3^ (vertical axis of the grid) and the size of the RDS sample is varied from 250 to 750 (horizontal axis of the grid). In each of the 12 graphs, the x-axis varies the average degree λ from 3 to 10. For each choice of λ, the medians and quartile ranges of *n*_3_ are given for each of the 5 graph families. Each of these is determined by 900 simulations (30 graphs times 30 uniformly drawn samples in each graph).

Fig 3 shows that the median of *n*_3_ converge to the true population size, much like the performance of the *n*_2_ estimator. In all the networks, the medians of *n*_3_ estimates are all very close to the their true network populations, regardless the sample size, population size, and type of network topology. In addition, Fig 3 shows that as sample size increases, the interquartile range of the estimates decreases. For example, when *n* = 5 ⋅ 10^3^ and *r* = 250, Lognormal degree distribution graphs with λ = 3 experience a interquartile range of 1915 in their *n*_3_ estimates (39.1% of the median). In comparison, when *r* = 750, the interquartile range for this family of graphs decreases to 604 (a 68.5% reduction). The magnitude of this effect decreases as networks grow larger. For example, in a network of size *n* = 40 ⋅ 10^3^, increasing the sample size from *r* = 250 to *r* = 750 causes the interquartile range of the *n*_3_ estimate to undergo a (still sizable) 55.0% decrease.

## 5 Subject privacy through hashing

Significant obstacles arise in the direct application of estimators *n*_1_, *n*_2_, *n*_3_ (see (8), (15), and (28), respectively). In many circumstances where RDS is used, researchers are often required to measure the sizes of stigmatized networked populations (e.g. people who inject drugs, sex workers, individuals engaged in specific types of illegal activity, etc.) and within social communities that naturally seek to remain “unidentified”. In these circumstances, the membership of sets *S* and *R*(*S*, *F*) is often not explicitly knowable because individuals are reluctant to unambiguously identify themselves or their social network peers.

To formalize and accommodate notions of privacy required under such circumstances within the estimation procedures described above, we assume that each individual in *V* = {*v*_1_, *v*_2_, …, *v*_|*V*|_} has a unique ID; for simplicity we take the ID of *v*_*i*_ ∈ *V* to be the integer *i* (for *i* = 1, …, |*V*|). Towards ensuring anonymity, we imagine a *hashing* [89] function *ψ*: *V* → Ω that assigns each individual’s ID to a code in Ω. We thus follow the general framework of Privatized Network Sampling (PNS) design [53], mimicking the hash functions of telefunken-type [50].

By taking *ψ* to be a random (not necessarily 1-to-1) function that is difficult to invert, subjects are convinced that disclosing the hash code of an individual does not unambiguously identify the individual themselves, and so preserves their privacy.

**Assumption 3**. *Suppose* *V* *is a set of individuals obtained via RDS referral tree F*. *While each* *v*_*i*_ ∈ *V* *is unwilling to disclose their own ID* *i*, *and is secretive about the IDs of their peers* {*j*|*v*_*j*_ ∈ *N*(*v*_*i*_, ∅)}, *they are readily willing to reveal (a) the own hash code* *ψ*(*v*_*i*_)*; (b) the (multiset of) hash codes of their peers (outside the referral tree F):*

$$N_{u}^{\psi }\left(S,F\right)≔\underset{{}_{\left(u,v\right)\notin F}^{v\in N\left(u\right)}}{∐}\left\{\psi \left(v\right)\right\}\subseteq \Omega$$ (36)

*and (c) their own network size* $d\left(v_{i}\right)=\langle N_{u}^{\psi }\left(S,F\right)\rangle$, *excluding the referral tree F*.

**Fig 4:**
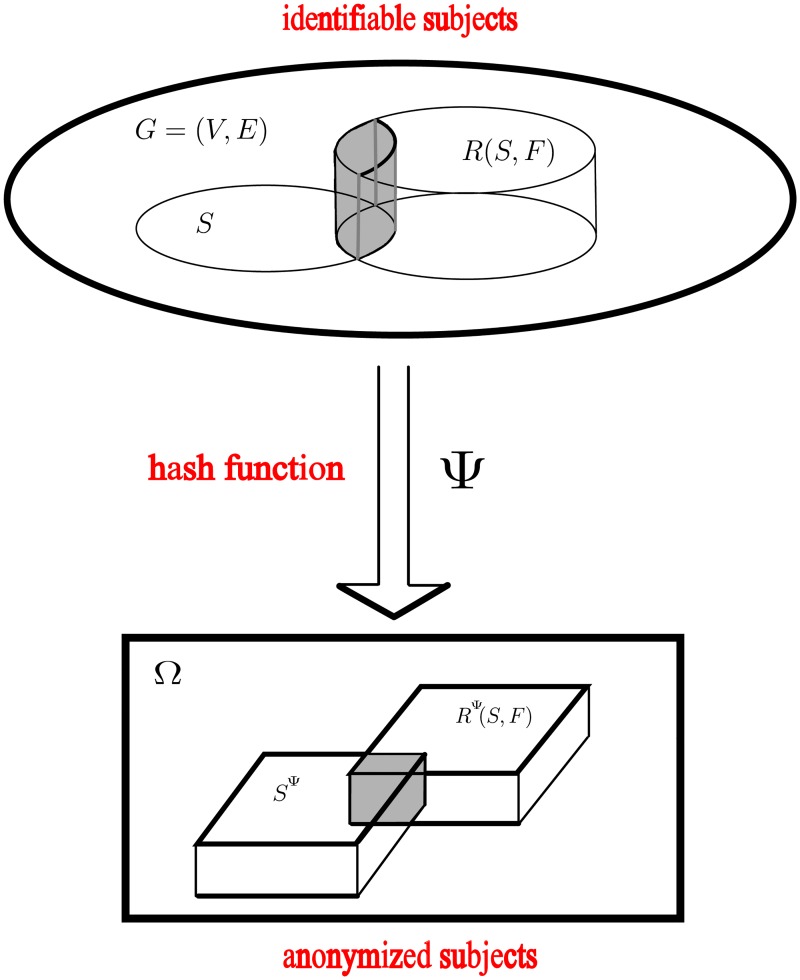
The action of *ψ* on *V*.

**Assumption 4**. *To simplify our analysis, throughout what follows, we will assume ψ is a function chosen uniformly at random from the space of all functions from V* → Ω. *We will refer to such a ψ as a “random hash function” from V to* Ω. *The action of ψ on the V is illustrated in* Fig 4. *In Section 6.3, we describe ways to translate the results of this paper to settings where ψ is not a uniformly random hashing function*.

In practice, *ψ*(*v*) might be an obtained by amalgamating a well-defined tuple of characteristics of *v* which are known to *v*’s friends (e.g. *v*’s gender, phone number, hair color, approximate age, racial category, etc.) and then encoding this using a cryptographic function. A related coding technique was used in our earlier work on estimating the size of the methamphetamine using population in New York City, where it was referred to as the *telefunken* code [50].

### 5.1 Revised estimators incorporating hashing

We begin by “lifting” the terms introduced in the earlier Definition 1, to the hashing or PNS framework [53].

**Definition 10**. *Let G* = (*V*, *E*) *be a graph, and ψ*: *V* → Ω *a random hash function*. *Let H* = (*S*, *F*) *be a subgraph on S* ⊆ *V* *with edge set F* ⊆ *E* ∩ (*S* × *S*). *The (multiset of) hash codes of the subjects is*

$$S^{\psi }≔\{\psi (v)\;|\;v\in S\}\subseteq \Omega .$$ (37)

*The ψ*-free ends *of S (in G modulo H) are taken to be the disjoint union (multiset)*

$$R^{\psi }(S,F)≔\underset{u\in S}{∐}N^{\psi }\left(u,F\right)\subseteq \Omega$$ (38)

*and the ψ*-matches *of (in G modulo H) are taken to be the disjoint union (multiset)*

$$M^{\psi }(S,F)≔\underset{u\in S}{∐}(N^{\psi }\left(u,F\right)\cap S^{\psi })\subseteq \Omega .$$ (39)

*We denote their respective multiset cardinalities as*

$$\begin{array}{ll} \langle R^{\psi }\left(S,F\right)\rangle ≔ & \sum_{u\in S}\left|N^{\psi }\left(u,F\right)\right|\\ \langle M^{\psi }\left(S,F\right)\rangle ≔ & \sum_{u\in S}\left|N^{\psi }\left(u,F\right)\cap S^{\psi }\right|. \end{array}$$

*The reader may wish to compare expressions* (36), (38), *and* (39) *with the non-hashed analogues in Definition 1’s expressions* (5), (6), *and* (7).

The next Lemma is foundational and justifies the proposed revised estimates $n_{1}^{\psi }$, $n_{2}^{\psi }$, and $n_{3}^{\psi }$, which will be presented subsequently.

**Lemma 2**. *Let G* = (*V*, *E*) *a graph with* |*V*| = *n*′, *sampled from the space of all* *n*′-*vertex graphs by configuration sampling with respect to degree distribution* D. *Let S* ⊆ *V* *be an RDS sample collected as a subgraph H* = (*S*, *F*) *be with edge set F* ⊆ *E* ∩ (*S* × *S*). *Let c* ≔ |*S*|/|*V*|, *where c* ≪ 1. *Accepting Assumption 1*, *take ψ*: *V* → Ω *to be a random hash function*.

1. *Suppose u* ∈ *S reports its own code x* ≔ *ψ*(*u*), *the code* *y* ≔ *ψ*(*v*) *of one of its neighbors v* ∈ *N*_*u*_(*S*, *F*). *If w* ∈ *ψ*^−1^(*y*) ∩ *S* *is selected uniformly at random, and w has degree d*(*w*), *then*
  $$Prob(w=v)=\frac{1}{\frac{n^{\prime }-1}{\left|\Omega \right|}\frac{\tilde{d}\left(S\right)}{\left(d(w)-1\right)}+1}.$$
2. *For each code y* ∈ Ω, *over the space of all random hash functions*,
  $$E\left[\left\langle M^{\psi }\left(S,F\right)\right\rangle \right]=\hat{m}\left(y,n^{\prime }\right)$$
  *where*
  $$\begin{array}{cc} \hat{m}(y,n^{\prime })≔ & \sum_{w\in \psi^{-1}\left(y\right)\cap S}\frac{1}{\frac{n^{\prime }-1}{\left|\Omega \right|}\frac{\tilde{d}\left(S\right)}{\left(d(w)-1\right)}+1}\\ \hat{m}(n^{\prime })≔ & \sum_{y\in M^{\psi }\left(S,F\right)}\hat{m}\left(y,n^{\prime }\right). \end{array}$$

*Proof*. (1) Because *ψ* is a random function, for any *z* ∈ Ω

$$E[|\psi^{-1}\left(z\right)|]=\frac{n^{\prime }}{\left|\Omega \right|}.$$

The expected total number of free ends incident to some vertex in the set *ψ*^−1^(*y*)\{*w*} is

$$\frac{\left(n^{\prime }-1\right)\left(1-c\right)}{\left|\Omega \right|}\cdot \tilde{d}\left(S\right)+\frac{(n^{\prime }-1)c}{\left|\Omega \right|}\cdot (\tilde{d}\left(S\right)-1)$$

and since *w* ∈ *S*, the expected number of free ends incident to *w* is *d*(*w*) − 1. So

$$Prob(w=v)=\frac{d(w)-1}{\frac{\left(n^{\prime }-1\right)\left(1-c\right)}{\left|\Omega \right|}\cdot \tilde{d}\left(S\right)+\frac{(n^{\prime }-1)c}{\left|\Omega \right|}\cdot (\tilde{d}\left(S\right)-1)+\left(d\left(w\right)-1\right)}.$$

dividing through by *d*(*w*) − 1, and considering *c* ∼ 0, the Lemma is proved. Assertion (2) follows from (1) by linearity of expectation.

**Definition 11**. *If* f:R→R *is a real-valued function defined on the reals, then we denote RootOf*^+^[*f*(*x*) = 0, *x*] *to be (any one of the positive “roots”)* $x^{*}\in R$ *that satisfies the condition* *f*(x*) = 0, *and* x* > 0.

**Definition 12**. *Given a graph G* = (*V*, *E*), *and* *ψ*: *V* → Ω *a random hash function*. *Fix S* ⊆ *V*, *and* *H* = (*S*, *F*) *a subgraph on* *S* ⊆ *V* *with edge set F* ⊆ *E* ∩ (*S* × *S*). *We define*

$$n_{2}^{\psi }(S,F)≔RootOf^{+}[f_{2}^{\psi }\left(n^{\prime },S,F\right)-n^{\prime }=0,\;\;n^{\prime }]$$ (40)

*where*

$$f_{2}^{\psi }(n^{\prime },S,F)≔\frac{\frac{\bar{d}\left(S\right)-1}{\tilde{d}\left(S\right)}\cdot \left\langle S^{\psi }\right\rangle \cdot \left\langle R^{\psi }\left(S,F\right)\right\rangle }{\hat{m}\left(n^{\prime }\right)}$$

*and RootOf*^+^ *is the root operation described in Definition 11*.

**Definition 13**. *Given a graph* *G* = (*V*, *E*), *a set* *S* ⊆ *V*, *and H* = (*S*, *F*) *a subgraph on* *S* ⊆ *V* *with edge set F* ⊆ *E* ∩ (*S* × *S*). *Let D* ⊆ *S* *satisfying* |*D*| > 1 *and*

$$s_{1}\neq s_{2}\Rightarrow C_{\gamma }\left(s_{1}\right)\cap C_{\gamma }\left(s_{2}\right)=\emptyset .$$

*Take γ*: *S* → *D* *as described in Definition 8*. *The (multiset of) hash codes of vertices in the component of u are denoted*

$$C_{\gamma }^{\psi }(u)≔\left\{\psi \left(v\right)\;|\;v\in C_{\gamma }\left(u\right)\right\}\subseteq S^{\psi }$$ (41)

*while the codes of the complement set (inside S) are written as*

$${\tilde{C}}_{\gamma }^{\psi }(u)≔\left\{\psi \left(v\right)\;|\;v\in {\tilde{C}}_{\gamma }\left(u\right)\right\}\subseteq S^{\psi }.$$

*Note that* $C_{\gamma }^{\psi }\left(u\right)\cap {\tilde{C}}_{\gamma }^{\psi }\left(u\right)$ *may be non-empty. For each seed s* ∈ *D*, *we define the* cross-seed *ψ*-matches *from* $C_{\gamma }^{\psi }\left(s\right)$ *in G modulo H as the disjoint union (multiset)*

$$X^{\psi }(s,F,\gamma )≔\underset{u\in C_{\gamma }\left(s\right)}{∐}(N^{\psi }\left(u,F\right)\cap {\tilde{C}}_{\gamma }^{\psi }\left(s\right))\subseteq \Omega .$$ (42)

*The reader may wish to compare expressions* (41) and (42) *with the non-hashed analogues in Definition 8’s expressions* (26) and (27). We also define

$$\begin{array}{cc} \tilde{x}(y,s,\gamma ,n^{\prime })≔ & \sum_{w\in \psi^{-1}\left(y\right)\cap {\tilde{C}}_{\gamma }\left(s\right)}\frac{1}{\frac{n^{\prime }-1}{\left|\Omega \right|}\frac{\tilde{d}\left(S\right)}{\left(d(w)-1\right)}+1}\\ \hat{x}(s,F,\gamma ,n^{\prime })≔ & \sum_{y\in X^{\psi }\left(s,F,\gamma \right)}\tilde{x}\left(y,s,\gamma ,n^{\prime }\right). \end{array}$$

**Definition 14**. *Given a graph G* = (*V*, *E*), *a set S* ⊆ *V*, *and H* = (*S*, *F*) *a subgraph on S* ⊆ *V* *with edge set F* ⊆ *E* ∩ (*S* × *S*). *We define*

$$n_{3}^{\psi }(S,F)≔RootOf^{+}[f_{3}^{\psi }\left(n^{\prime },S,F,D,\gamma \right)-n^{\prime }=0,\;\;n^{\prime }]$$ (43)

*where*

$$f_{3}^{\psi }(n^{\prime },S,F,D,\gamma )≔\frac{\sum_{s\in D}\frac{\bar{d}\left({\tilde{C}}_{\gamma }\left(s\right)\right)-1}{\tilde{d}\left(S\right)}\cdot \left\langle {\tilde{C}}_{\gamma }^{\psi }\left(s\right)\right\rangle \cdot \left\langle R^{\psi }\left(C_{\gamma }\left(s\right),F\right)\right\rangle }{\sum_{s\in D}\hat{x}\left(s,F,\gamma ,n^{\prime }\right)}$$

*and RootOf*^+^ *is the root operation described in Definition 11*.

### 5.2 Evaluating $n_{2}^{\psi }$ on synthetic networks

The experiments discussed here follow the framework used in prior experiments described above. Samples are derived using the RDS process operating as specified in Assumption 2. The hash space size used for the encoding of each agent’s identity was varied from |Ω| = 2 ⋅ 10^3^ to 256 ⋅ 10^3^.

**Fig 5:**
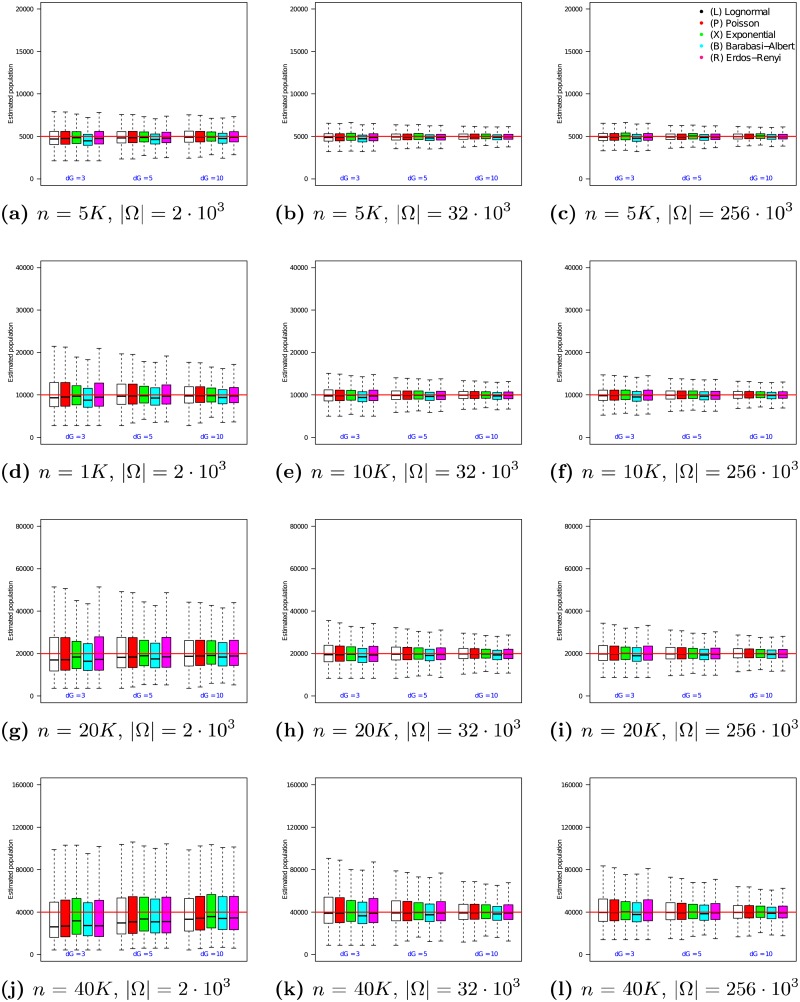
Estimator $n_{2}^{\psi }$ on RDS samples of size *r* = 500 with |Ω| = 2 ⋅ 10^3^ to 256 ⋅ 10^3^. In each box, the thick line indicates the sample median; the top of the box is the median of the upper half of the estimated values (75% quartile); the bottom of the box indicates the median of the lower half of the estimated values (25% quartile); and the whiskers indicate the full range of estimated values. No (finite) outliers were removed.

The 12 graphs in Fig 5 present the performance of the $n_{2}^{\psi }$ estimator as the true population size *n* is varied from 5 ⋅ 10^3^ to 40 ⋅ 10^3^ (vertical axis of the grid), the sample size is fixed to *r* = 500 and the hash space size was varied from |Ω| = 2 ⋅ 10^3^ to 256 ⋅ 10^3^ (horizontal axis of the grid). In each of the 12 graphs, the x-axis varies the average degree λ from 3 to 10. For each choice of λ, the medians and quartile ranges of $n_{2}^{\psi }$ are given for each of the 5 graph families. Each of these is determined by 900 simulations (30 graphs times 30 uniformly drawn samples in each graph).

Fig 5 shows that as hash space size increases, the medians of $n_{2}^{\psi }$ converge to the true population size. For example, when *n* = 5 ⋅ 10^3^ and |Ω| = 2 ⋅ 10^3^, Lognormal degree distribution graphs with λ = 3 have a median $n_{2}^{\psi }$ value of 4705 (a 5.9% offset from the true value of *n* = 5 ⋅ 10^3^). In comparison, when |Ω| = 256 ⋅ 10^3^, the median value for this family of graphs is 4901 (just 2.0% offset from the true value). As the hash space size increases from |Ω| = 2 ⋅ 10^3^ to |Ω| = 256 ⋅ 10^3^, the error in the median estimate decreases by 3.9%. The magnitude of this phenomenon increases as networks grow larger. For example for a network of size *n* = 40 ⋅ 10^3^, increasing the hash space size from |Ω| = 2 ⋅ 10^3^ to |Ω| = 256 ⋅ 10^3^ causes the error in the median $n_{2}^{\psi }$ estimate to undergo a 33.9% change.

In addition, Fig 5 shows that as hash space size increases, the interquartile range of the estimates decreases. For example, when *n* = 5 ⋅ 10^3^ and |Ω| = 2 ⋅ 10^3^, Poisson degree distribution graphs with λ = 3 experience a interquartile range of 1522 in their $n_{2}^{\psi }$ estimates (32.0% of the median). In comparison, when |Ω| = 256 ⋅ 10^3^, the interquartile range for this family of graphs decreases to 793 (a 47.9% reduction). The magnitude of this effect increases as networks grow larger. For example for a network of size *n* = 40 ⋅ 10^3^, increasing the hash space size from |Ω| = 2 ⋅ 10^3^ to |Ω| = 256 ⋅ 10^3^ causes the interquartile range of the $n_{2}^{\psi }$ estimate to undergo a 42.1% decrease.

### 5.3 Evaluating $n_{3}^{\psi }$ on synthetic networks

A second set of experiments shows the performance of the $n_{3}^{\psi }$ performance under identical hashing conditions used to test $n_{2}^{\psi }$. These experiments also follow the framework described in Section 4.2 and use samples derived from an RDS process operating as specified in Assumption 2. The hash space size was varied from |Ω| = 2 ⋅ 10^3^ to 256 ⋅ 10^3^.

**Fig 6:**
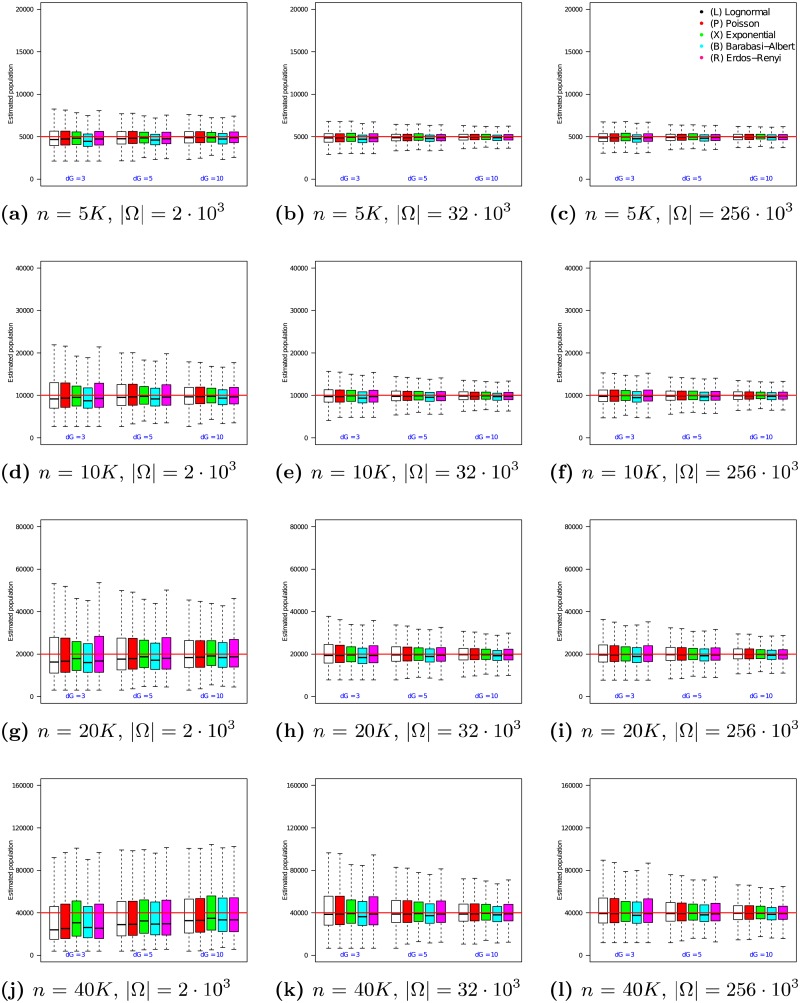
Estimator $n_{3}^{\psi }$ on RDS samples of size *r* = 500 with |Ω| = 2 ⋅ 10^3^ to 256 ⋅ 10^3^. In each box, the thick line indicates the sample median; the top of the box is the median of the upper half of the estimated values (75% quartile); the bottom of the box indicates the median of the lower half of the estimated values (25% quartile); and the whiskers indicate the full range of estimated values. No (finite) outliers were removed.

The 12 graphs in Fig 6 present the performance of the $n_{3}^{\psi }$ estimator as the true population size *n* is varied from 5 ⋅ 10^3^ to 40 ⋅ 10^3^ (vertical axis of the grid), the sample size is fixed to *r* = 500 and the hash space size was varied from |Ω| = 2 ⋅ 10^3^ to 256 ⋅ 10^3^ (horizontal axis of the grid). In each of the 12 graphs, the x-axis varies the average degree λ from 3 to 10. For each choice of λ, the medians and quartile ranges of $n_{3}^{\psi }$ are given for each of the 5 graph families. Each of these is determined by 900 simulations (30 graphs times 30 uniformly drawn samples in each graph).

Fig 6 shows that as hash space size increases, the medians of $n_{3}^{\psi }$ converge to the true population size. For example, when *n* = 5 ⋅ 10^3^ and |Ω| = 2 ⋅ 10^3^, Lognormal degree distribution graphs with λ = 3 have a median $n_{3}^{\psi }$ value of 4667 (a 6.7% offset from the true value of *n* = 5 ⋅ 10^3^). In comparison, when |Ω| = 256 ⋅ 10^3^, the median for this family of graphs is 4865 (just 2.7% offset from the true value). As the hash space size increases from |Ω| = 2 ⋅ 10^3^ to |Ω| = 256 ⋅ 10^3^, the error in the median estimate decreases by 4.0%. The magnitude of this phenomenon increases as networks grow larger. For example for a network of size *n* = 40 ⋅ 10^3^, increasing the hash space size from |Ω| = 2 ⋅ 10^3^ to |Ω| = 256 ⋅ 10^3^ causes the error in the median $n_{3}^{\psi }$ estimate to undergo a 38.4% change.

In addition, Fig 6 shows that as hash space size increases, the interquartile range of the estimates decreases. For example, when *n* = 5 ⋅ 10^3^ and |Ω| = 2 ⋅ 10^3^, Exponential degree distribution graphs with λ = 3 experience a interquartile range of 1491 in their $n_{3}^{\psi }$ estimates (31.0% of the median). In comparison, when |Ω| = 256 ⋅ 10^3^, the interquartile range for this family of graphs decreases to 905 (a 39.3% reduction). The magnitude of this effect increases as networks grow larger. For example for a network of size *n* = 40 ⋅ 10^3^, increasing the hash space size from |Ω| = 2 ⋅ 10^3^ to |Ω| = 256 ⋅ 10^3^ causes the interquartile range of the $n_{3}^{\psi }$ estimate to undergo a 43.0% decrease.

## 6 Impacts of non-uniformity

The experiments described in previous sections of this paper assumed an RDS process that begins with a set of seeds *D* ⊆ *V* sampled *uniformly at random without replacement*. More precisely, *D* = *X*_|*D*|_ is the last entry in sequence *X*_0_, *X*_1_, …*X*_|*D*|_, where *X*_0_ = ∅ and *X*_*i*_ = *X*_*i*−1_ ∪ {*u*_*i*_} with

$$Pr\left(u_{i}=u\right)=\{\begin{array}{cc} \frac{1}{\left|V|-\right|X_{i-1}|} & u\in V\backslash X_{i-1}\\ 0 & \text{otherwise} \end{array}$$ (44)

for each *u* ∈ *V*. While the uniform model allowed formal analysis of the estimators’ properties to be tractable, many researchers have noted that practical deployments of RDS often exhibit bias in seed selection [90–92]. This bias originates in local features of the network topology (e.g. variation in node degrees) as well as global properties (e.g. the presence of community structures).

### 6.1 Degree-biased selection of RDS seeds

We begin by describing experimental findings on the differential impacts of degree-based bias in initial seed selection on the performance of the $n_{3}^{\psi }$ estimator. Towards this, we define a new model of seed selection in which a real-valued parameter ρ∈R controls degree-based bias. In particular, expression (44) is generalized to

$$Pr\left(u_{i}=u\right)=\{\begin{array}{cc} \frac{e^{\rho \cdot d(u)}}{\sum_{v\in V\backslash X_{i-1}}e^{\rho \cdot d(v)}} & u\in V\backslash X_{i-1}\\ 0 & \text{otherwise} \end{array}$$ (45)

for each *u* ∈ *V*. Note that when *ρ* = 0 expression (45) reduces to the uniform random selection of seeds prescribed in (44). When *ρ* > 0, seed selection is biased towards the network’s high degree vertices; when *ρ* < 0, low degree vertices are favored.

The first segment of Table 1 shows that as *ρ* is varied between -1 and +1, non-uniform seed selection has no discernable negative differential impact on the performance of RDS estimator $n_{3}^{\psi }$. While the data in the first segment of Table 1 are based on 30 RDS samples (*r* = 500) on each of 30 graphs from $L(\lambda =5,n=10^{4})$, i.e. graphs with 10*K* nodes and a Lognormal degree distribution as described in Section 4.1, the conclusion for the other 5 graph families is similar.

**Table 1:** Varying seed selection bias (*n* = 10*K*, *r* = 500, |Ω| = 256 ⋅ 10^3^, $\bar{d}\left(V\right)=5$).

	*K*	*ρ*	*μ*	$n_{3}^{\psi }$ median	$n_{3}^{\psi }$ I.Q.R.
*ρ*: Seed selection bias	1	-1	N.A.	9966.3	2253.0
		-0.5		10104.7	2374.9
		-0.4		10057.2	2313.4
		-0.3		9956.4	2267.2
		-0.2		9981.6	2231.6
		-0.1		9868.0	2254.3
		0		9909.0	2170.4
		0.1		9903.2	2155.1
		0.2		9963.4	2271.6
		0.3		9766.7	2277.9
		0.4		9921.4	2170.2
		0.5		9942.7	2307.1
		1		9793.4	2165.6
*K*: Number of components	1	0	0.5	10070.9	2325.5
	2			9977.7	2220.8
	4			9797.8	2136.7
	8			9312.6	2216.2
	16			8373.9	1900.0
*μ*: Cross component probability	8	0	0.1	3088.4	1102.6
			0.2	5526.1	1864.7
			0.3	7595.0	1961.6
			0.4	8639.9	2010.4
			0.5	9345.6	2033.6

### 6.2 Community structures

Next we consider the impact of community structures which can potentially create bottlenecks for RDS and restrict the reach of subject’s self-reported ego networks [91, 92]. We quantify the impacts of such structures on the $n_{3}^{\psi }$ estimator through simulation experiments, and towards this, extend each of the 5 families defined in Section 4.1 to support the controlled presence of community effects. Two new parameters are introduced: the number of communities *K*, and the cross-community connection probability *μ*. The space L(λ,n), for example, is thus extended to a space L(λ,n,K,μ) consisting of graphs of size *K* ⋅ ⌊*n*/*K*⌋, i.e. approximately *n*, which is sampled from as follows:

1. Sample *K* graphs *G*_1_ = (*V*_1_, *E*_1_), …*G*_*k*_ = (*V*_*k*_, *E*_*k*_) from L(λ,n) as defined in Section 4.1. Define $V\infty_{i=1}^{K}V_{i}$ to be the vertex set of our sampled graph. Take $E=\infty_{i=1}^{K}E_{i}$ to be our initial approximation of the edge set of our sampled graph, to be updated according to the rewiring process below.
2. For each *i* ∈ {1, 2, …, *K*}, and each *u* ∈ *V*_*i*_, with probability *μ*:
  - Choose *j* ∈ {1, 2, …, *K*}\{*i*} uniformly at random, and then choose *v* ∈ *V*_*j*_ uniformly at random.
  - Choose *u*′ ∈ *N*(*u*) ∩ *V*_*i*_ uniformly at random.
  - Choose *v*′ ∈ *N*(*v*) ∩ *V*_*j*_ uniformly at random.
  - Modify *E* by removing (*u*, *u*′) and (*v*, *v*′) from *E*.
  - Modify *E* by adding (*u*, *v*) and (*u*′, *v*′) to *E*.
3. Completion of step (2) yields the sampled graph (*V*, *E*) on *K* ⋅ ⌊*n*/*K*⌋ vertices, having *K* communities each coming from family L(λ,n) and wired together so that roughly *μ* fraction of each community’s members has a connection to some member of a different community (and the degree distribution of the graph as a whole is consistent with the bias of family L).

The families P(λ,n,K,μ),X(λ,n,K,μ),B(λ,n,K,μ), and E(λ,n,K,μ), are defined analogously. When *μ* ∼ 1 or *K* ∼ 1, community effects are insignificant. As *μ* → 0^+^ or *K* ≫ 1, the population consists of many effectively isolated communities. Whenever a set of seeds are to be selected from the network (e.g. to obtain a respondent driven sample), all seeds are chosen (uniformly at random) *from community 1*.

The second segment of Table 1 shows that as *K* is increased from 1 to 16 (while *μ* is held fixed at 0.5), increasing the number of communities causes $n_{3}^{\psi }$ to slightly underestimate population size. For example, when the network consists of *K* = 8 communities, a median estimate falls short of the true value by 7%; for *K* = 16 communities the deficit becomes 16%. The third and final segment of Table 1 shows that as *μ* is decreased from 0.5 to 0.1 (while *K* is held fixed at 8), increasing community isolation causes $n_{3}^{\psi }$ to significantly underestimate population size. For example, when the inter-community connection probability *μ* = 0.4 the deficit is 14%, but when *μ* = 0.2 the estimate produced is roughly 45% of the true value. While the data in the second and third segments of Table 1 are based on 30 trials on each of 30 graphs from L(λ,n), i.e. graphs with Lognormal degree distribution as described in Section 4.1, the results for the other 5 graph families are quite similar.

### 6.3 Non-uniform hash functions

The experiments and analyses so far have considered a uniform random hashing function *ψ*, and have shown that the size of the hash space |Ω| has a significant impact on estimator variance. The uniform hashing assumption is reasonable when each individual’s anonymity-preserving code is based on attributes that have been uniformly randomly assigned across the population. For example, it is reasonable to expect that a telephone company will assign numbers to customers randomly, and thus a code that is built from the parity and scale of the final 4 digits of each individual’s phone number would constitute a uniform random hash function.

In this section, we describe how to translate the conclusions of previous experiments and analyses to settings where the hashing function is not uniformly random. This would likely be the case if *ψ* were built from each individual’s demographic characteristics (e.g. age, height, hair color, and race) that are known to vary non-uniformly across the population. For example, if subjects and reports were encoded using 4 categories for age, 3 categories for height, 3 for hair color, and 5 categories for race, one could only say that the hash space size was 4 × 3 × 3 × 5 = 180 if *all combinations of these attributes were equally likely* to appear. Researchers employing such non-uniform hashing functions may want to know the *equivalent* uniform hash space size |Ω|, so as to correctly translate the results of previous sections into reasonable expectations for the non-uniform situation at hand. The following Lemma will assist in defining this translation:

**Lemma 3**. *Let A, B be finite sets, and ψ*: *A* → *B be a uniformly random function. Then*

$$E|\psi \left(A\right)|=\left|B\right|\cdot [1-(1-\frac{1}{\left|B\right|}{)}^{\left|A\right|}].$$

*Proof*. We seek the expected number of distinct items obtained in sampling |*A*| elements from *B* with replacement. Consider *x* ∈ *B*, then

$$Pr\left(\left\{x\in \psi \left(A\right)\right\}\right)=1-Pr\left(\left\{x\notin \psi \left(A\right)\right\}\right)=1-(1-\frac{1}{\left|B\right|}{)}^{\left|A\right|}.$$

The result then follows by linearity of expectation.

**Proposition 4**. *Let A, B be finite sets, and ψ*: *A* → *B* *a uniformly random function. Suppose* |*A*| = *x* *and* |*ψ*(*A*)| = *y*, *where* *x*, *y* ≫ 0, *then the maximum likelihood estimator of* |*B*| *is given by*

$$\left|B\right|=\frac{xy}{y\cdot W(-\frac{x}{y}\left(e^{-\frac{x}{y}}\right))+x}$$ (46)

*where Lambert’s W function is the inverse function of f*(*W*) = *We*^*W*^.

*Proof*. Applying Lemma 3, the maximum likelihood estimator is obtained by solving

$$y=\left|B\right|\cdot (1-(1-\frac{1}{\left|B\right|}{)}^{x})$$ (47)

for |*B*|. Since |*B*| ≥ *y* ≫ 0, we may approximate

$$\text{log}(\frac{|B|-1}{\left|B\right|})\approx -\frac{1}{\left|B\right|}$$

when |*B*| is large. Then we have

$$1-(1-\frac{1}{\left|B\right|}{)}^{x}=1-\text{exp}(x\;\text{log}(\frac{|B|-1}{\left|B\right|}))\approx 1-\text{exp}(-\frac{x}{\left|B\right|}).$$

Eq (47) now becomes

$$y=\left|B\right|\cdot (1-\text{exp}(-\frac{x}{\left|B\right|})),$$

which when solved for |*B*| yields expression (46) above.

Proposition 4 tells us that the image of a set of size *x* is expected to have size *y*, provided the function is a uniform random map into a set whose size is given by expression (46). Such a combinatorial result can be used to compute the equivalent uniform hash space size in settings where the hash function is non-uniform. In particular, if we have *x* = |*A*| subjects, who provide us with exactly *y* = |*ψ*(*A*)| distinct codes (*y* ≤ *x*), then the equivalent uniform hash space size |Ω| is given by expression (46) above.

## 7 Evaluating estimators on real networks

While a range of degree distributions and randomly occurring clusterings can be expected in idealized topologies, the performance of RDS-based estimators $n_{2}^{\psi }$ and $n_{3}^{\psi }$ on organically arising, natural human networks may vary. To test this possibility, we perform a number of random-start, RDS-based estimation experiments on the Brightkite data set. Brightkite was once a location-based social networking service provider where users shared their locations by checking-in. The friendship network was collected using their public API, and consists of |*V*| = 58,228 nodes and |*E*| = 214,078 edges [93]. Though originally a directed graph, we symmetrized the edges for the purposes of these experiments. Since not all users made a public check-in during the data collection period, the population we used here consists of 51,406 people. The average clustering coefficient in the network was 0.1723, while the fraction of closed triangles is 0.03979. The diameter (longest-shortest path in the symmetrized network) is 16, though the 90-percentile effective diameter is 6.

**Fig 7:**
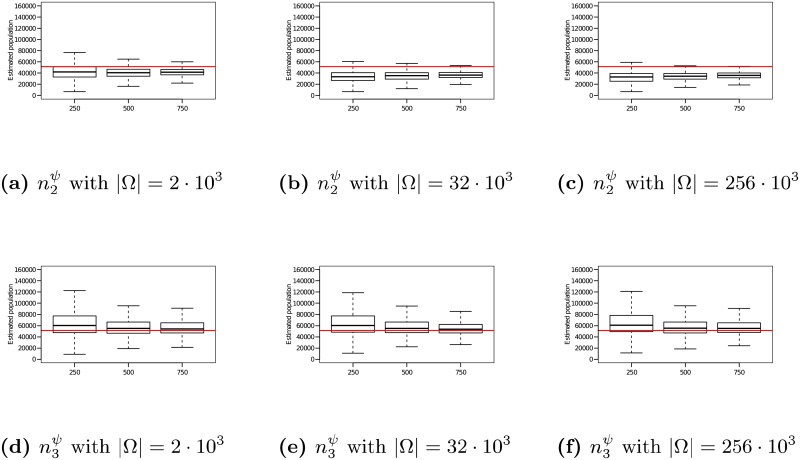
Estimator $n_{2}^{\psi }$ (above) and $n_{3}^{\psi }$ (below) on Brightkite network; |Ω| = 2 ⋅ 10^3^ to 256 ⋅ 10^3^, with sample size *r* = 250, 500, 750. In each box, the thick line indicates the sample median; the top of the box is the median of the upper half of the estimated values (75% quartile); the bottom of the box indicates the median of the lower half of the estimated values (25% quartile); and the whiskers indicate the full range of estimated values. Data points that exceeded the third quartile boundary by over 1.5 times the interquartile range (IQR) were treated as outliers and removed.

For purposes of the experiment we generated 900 respondent-driven samples of size *r* = 250, 500, 750 and hash space size from |Ω| = 2 ⋅ 10^3^ to |Ω| = 256 ⋅ 10^3^ within the Brightkite network, each obtained via an RDS process operating as specified in Assumption 2. The boxplot graphs in Fig 7(a)–7(c) show that estimator $n_{2}^{\psi }$—where no accommodation is made for the tendency of RDS to oversample tightly clustered network neighborhoods—underestimates the true population size of 51,406 in every case. Given the high clustering coefficient of the network (17.2%), it seems likely that, for a given sampling tree, the peer-discovery process necessarily walks across close pairs of nodes that shared one or more common vertices. Of note is that increasing the sample size and hash space size does little to correct for these effects.

Graphs (d-f) in Fig 7 present the boxplots of Brightkite population estimates using estimator $n_{3}^{\psi }$. As above, we generated 900 respondent-driven samples of size *r* = 250, 500, 750 and hash space size from |Ω| = 2 ⋅ 10^3^ to |Ω| = 256 ⋅ 10^3^ within the Brightkite network. We see that the three different hash space sizes show similar results, while increasing the sample size *r* from 250 to 500 and 750 improves the accuracy of the median estimate. Unlike the case in Fig 7(a)–7(c), we don’t see a consistent pattern of underestimation, indicating that the cross-seed estimator $n_{3}^{\psi }$ was successful in compensating for the clustering found in the network. As above, the overall size of the hash space has minimal effect on the accuracy of the median estimate, but we note that an increase in the RDS sample size improves the accuracy of the median estimate and produces smaller interquartile ranges.

## 8 Discussion

The results shown here indicate that size estimates for hidden and hard-to-reach populations can be derived from RDS samples across a range of topologies, and in the presence of significant network clustering. As important, this is accomplished under conditions of anonymity by way of identity hashing, e.g. using telefunken codes [50] or a Privatized Network Sampling (PNS) design [53]. The $n_{3}^{\psi }$ estimator joins other successful, RDS-based population estimation procedures, such as those by Handcock and Gile [85], and Crawford, Wu, and Heimer [35]. Like Crawford et al, we make use of half-edge counts. However, our estimator invokes a different strategy—beginning with the original capture-recapture concept—and is shown to be robust across a wide range of topologies and assumptions.

A notable feature of the $n_{3}^{\psi }$ estimator is that a lower level of variance can be expected at conventional RDS sample sizes. For *r* = 500 to 750, interquartile ranges were low relative to both the median estimate and true population size (See segments 1 and 2 of Table 2 which summarize a slice of the data in Fig 6).

**Table 2:** A cross-section of the experimental findings in this paper.

	*n*	*r*	|Ω|	$\bar{d}\left(V\right)$	$n_{3}^{\psi }$ median	$n_{3}^{\psi }$ I.Q.R.
*n*: Population size	5,000	750	256 ⋅ 10^3^	10	4934.3	342.1
	10,000				9927.2	1068.6
	20,000				20018.7	2731.1
	40,000				39964.7	9621.1
*r*: Sample size	5,000	250	256 ⋅ 10^3^	10	4972.7	1080.8
		500			4957.0	501.6
		750			4934.3	342.1
|Ω|: Hash space size	5,000	750	2 ⋅ 10^3^	10	4875.8	945.1
			32 ⋅ 10^3^		4938.4	363.1
			256 ⋅ 10^3^		4934.3	342.1
$\bar{d}\left(V\right)$: Average degree	5,000	750	256 ⋅ 10^3^	3	4797.5	848.5
				5	4867.7	565.5
				10	4934.3	342.1

Additionally, when hashing was employed towards ensuring subject anonymity, sufficiently large hash spaces (32 ⋅ 10^3^ or larger) and samples sizes (500 or above) produced a narrow range of estimates (See segment 3 of Table 2 which summarizes a slice of the data in Fig 6). Given concerns about RDS sample variance generally [28], these results indicate robustness against the faults of a single sample.

Another consistent feature observed in these experiments is the performance of the $n_{3}^{\psi }$ estimator as graph density increases (See segment 4 of Table 2 which summarizes a slice of the data in Fig 6). In terms of the interquartile ranges, the estimator exhibits worse performance in sparse (i.e. $\bar{d}\left(V\right)=3$) as opposed to dense networks (i.e. $\bar{d}\left(V\right)=10$). Given the edge-sampling focus of our approach, this is not surprising. Fewer total edges suggest fewer total “matches” to discover, leading to greater variability depending on stochastic factors likely associated with the selection of RDS seeds and the random walk features of the RDS sampling process. These results suggest limits on the implementation of $n_{3}^{\psi }$ estimator in sparse graphs.

As researchers increasingly turn to RDS methods for sampling hard-to-reach populations, these results should be of considerable interest to those concerned with what is often referred to as “the denominator problem”. Where agencies and government administrations seek to understand both the scope of public health challenges and to measure the effectiveness of their intervention and promotion efforts, the ability to estimate population size (and with this, population prevalence) is widely needed. The results presented here indicate that “one step” methods are capable of providing such estimates. Along with the methods mentioned above, this work has the potential to provide public health officials and planners with means to more effectively promote the health of hidden populations—and thus the health of the larger populations in which they are embedded.

### 8.1 Limitations

**Fig 8:**
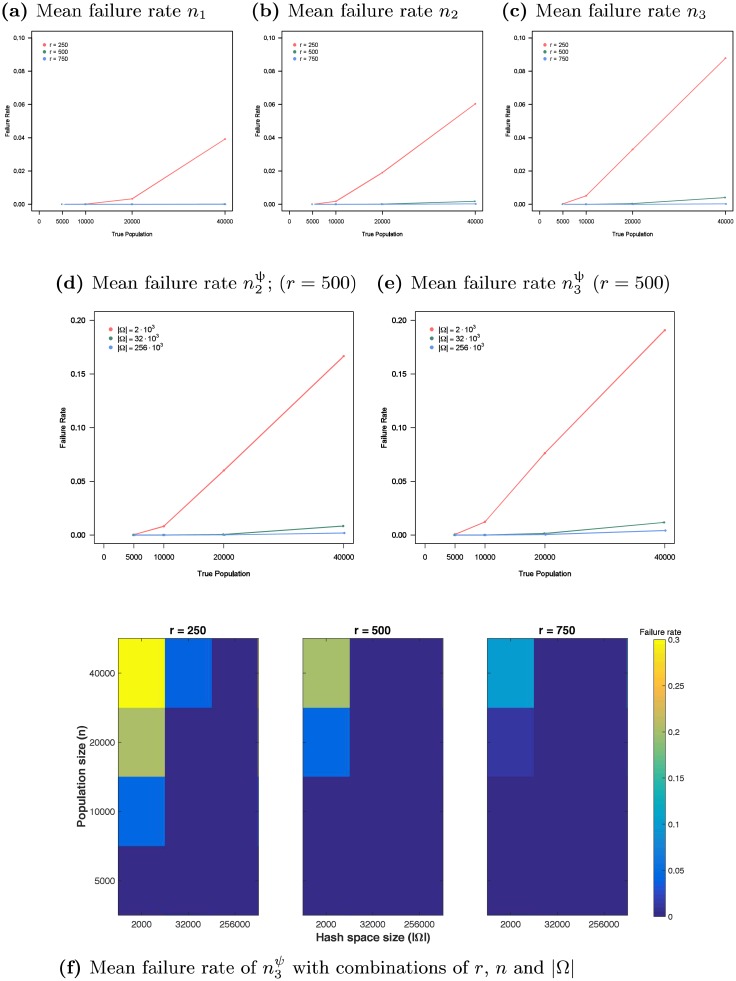
Mean failure rate analysis of the proposed estimators.

In using uniform random samples to estimate population size, it is possible for the proposed *n*_1_ estimator to “fail” if one finds that 〈*M*(*T*, ∅)〉 = 0 in Definition 3. This happens with greater frequency as the sample size *r* ≪ *n* the population size. Fig 8(a) shows the mean failure rate (the fraction of the 13,500 trials where *n*_1_ failed to produce a population estimate), for each choice of population size *n* (ranging from 5 ⋅ 10^3^ to 40 ⋅ 10^3^), and uniform sample size *r* (chosen to be 250, 500 or 750). We see from Fig 8(a) that the failure rate is non-linear in both *r* and *n*. For small uniform samples *r* = 250, the failure rate of *n*_1_ is ∼0 when *n* = 10 ⋅ 10^3^, but undergoes an inflection at *n* = 20 ⋅ 10^3^, and rises to 3.9% when the population size again doubles to *n* = 40 ⋅ 10^3^. Note that we considered each of 5 families L(λ,n),P(λ,n),X(λ,n),B(λ,n), and E(λ,n) defined in Section 4.1, and each λ = 3, 5, 10; from each of these 15 concrete sample spaces, we used configuration graph sampling to select 30 random graphs of size *n*. In each of these 5 × 3 × 30 = 450 graphs, we generated 30 uniform samples (for *n*_1_). In this manner, a total of 450 × 30 = 13,500 simulations were conducted.

Similarly, in using respondent-driven sampling to estimate population size, it is possible for the proposed *n*_2_ (resp. *n*_3_) estimators to “fail” if one finds that 〈*M*(*S*, *F*)〉 = 0 in Definition 4 (resp. ∑_*s*∈*D*_〈*X*(*s*, *F*, *γ*)〉 = 0 in Definition 9). Fig 8(b) shows the mean failure rate (the fraction of the 13,500 trials where *n*_2_ failed to produce a population estimate), for each choice of population size *n* (ranging from 5 ⋅ 10^3^ to 40 ⋅ 10^3^), and RDS sample size *r* (chosen to be 250, 500 or 750). RDS samples of size *r* = 250 exhibit an *n*_2_ failure rate of ∼0 when *n* = 5 ⋅ 10^3^, but undergo an inflection at *n* = 10 ⋅ 10^3^; the mean failure rate rises to 6% when the population size again doubles to *n* = 40 ⋅ 10^3^. In examining the *n*_3_ estimator, Fig 8(c) shows us that when it is used with RDS samples of size *r* = 250, it exhibits a failure rate of ∼0 when *n* = 5 ⋅ 10^3^, but the failure rate undergoes an inflection at *n* = 10 ⋅ 10^3^, rising to 8.8% when the population size again doubles to *n* = 40 ⋅ 10^3^. For sample sizes that are 2*X* and 3*X* as large (i.e. *r* = 500 and *r* = 750) the inflection point is not yet reached at *n* = 40 ⋅ 10^3^ and mean failure rates remain below 0.1%. This indicates that our estimators based on RDS are more robust against failure than the *n*_1_ uniform sampling estimator, and at typical RDS sample sizes (500 ≤ *r* ≤ 750), they are robust enough to be used in settings where the population size is expected to be on the order of *n* ∼ 40 ⋅ 10^3^.

Fig 8(d)–8(e) explore the impact of hash space size on the mean failure rate. Here we consider a fixed sample size *r* = 500 and vary the size of hash space |Ω| between 2 ⋅ 10^3^ and 256 ⋅ 10^3^. We observe that the mean failure rates of $n_{2}^{\psi }$ and $n_{3}^{\psi }$ (again taken across 13,500 experiments) grow linearly as *n* increases, but that the rate of growth depends on |Ω|. In particular, when |Ω| is too small (in this case 2 ⋅ 10^3^ or smaller), the mean failure rate is seen to grow steeply, even for small networks. The two graphs (d-e) make evident the tradeoff between subject anonymity/privacy and the failure rates of the estimator. When the hash space size is sufficiently large (32 ⋅ 10^3^−256 ⋅ 10^3^), failure rates remain low, but smaller hash spaces (which provide for greater anonymity) may produce greater instability in the estimators. Finally, the three heatmaps in Fig 8(f) show how the failure rate of $n_{3}^{\psi }$ rises whenever the hash space size or sample size decreases.

Although 32 ⋅ 10^3^ − 256 ⋅ 10^3^ may appear to be a very large hash space size, we note

$$\begin{array}{c} 10^{4}\leq 32\cdot 10^{3}\leq 10^{5}\leq 256\cdot 10^{3}\leq 10^{6}. \end{array}$$

Thus, asking research subjects for the last 5 or 6 digits of their own telephone number and those digits of their friends’ phone numbers would be sufficient to provide an accurate estimate (assuming that numerical digits are randomly assigned by phone service providers). In the event that research subjects remain reluctant to reveal precise digits of their own or their alter’s phone numbers, a telefunken code could be constructed [50] or a Privatized Network Sampling (PNS) design [53] employed.
